# Endothelial Nitric Oxide Synthase (eNOS) and the Cardiovascular System: in Physiology and in Disease States

**Published:** 2022-01-04

**Authors:** N Tran, T Garcia, M Aniqa, S Ali, A Ally, SM Nauli

**Affiliations:** 1 Arkansas College of Osteopathic Medicine, Fort Smith, AR, USA; 2 Chapman University and University of California, Irvine, CA, USA

**Keywords:** Nitric Oxide, Arteriosclerosis, Hypertension, Stroke, Myocardial Infarction, Exercise Pressor Reflex, Baroreflex, Ventrolateral Medulla, Nociception, ENOS polymorphism, ENOS gene, Vasodilatation, Glutamate, Blood Pressure, Sympa-thetic Nervous System, Cardiovascular Reflex, Endothelium, Angiotensin, Cyclic GMP, ENOS Dimerization

## Abstract

Endothelial nitric oxide synthase (eNOS) plays a critical role in regulating and maintaining a healthy cardiovascular system. The importance of eNOS can be emphasized from the genetic polymorphisms of the eNOS gene, uncoupling of eNOS dimerization, and its numerous signaling regulations. The activity of eNOS on the cardiac myocytes, vasculature, and the central nervous system are discussed. The effects of eNOS on the sympathetic autonomic nervous system (SANS) and the parasympathetic autonomic nervous system (PANS), both of which profoundly influence the cardiovascular system, will be elaborated. The relationship between the eNOS protein with cardiovascular autonomic reflexes such as the baroreflex and the *Exercise Pressor Reflex* will be discussed. For example, the effects of endogenous nitric oxide (NO) are shown to be mediated by the eNOS protein and that eNOS-derived endothelial NO is most effective in regulating blood pressure oscillations via modulating the baroreflex mechanisms. The protective action of eNOS on the CVS is emphasized here because dysfunction of the eNOS enzyme is intricately correlated with the pathogenesis of several cardiovascular diseases such as hypertension, arteriosclerosis, myocardial infarction, and stroke. Overall, our current understanding of the eNOS protein with a focus on its role in the modulation, regulation, and control of the cardiovascular system in a normal physiological state and in cardiovascular diseases are discussed.

## Introduction

Previous reviews have summarized how a reduced expression of the endothelial nitric oxide synthase (eNOS) enzyme and subsequent decreased production of NO result in an increased susceptibility/risk to develop essential hypertension [1,2], preeclampsia [3], diabetic nephropathy [4], retinopathy [5], migraine [6], and erectile dysfunction [7]; the role of the eNOS enzyme, including its modulation, regulation, and relevance within the cardiovascular system (CVS) in both normal physiological state and in several important cardiovascular diseases is elaborated. In addition, because the amount of NO generated by the vascular endothelial cells plays critical roles in the regulation and maintenance of the vascular tone, migration, production, proliferation, and maturation of cells, leukocyte adhesion, and platelet aggregation, it can be implied that eNOS is indeed an essential molecule for a properly functioning and healthy CVS, thus emphasizing the protective action of eNOS on the CVS.

## Endothelial Nitric Oxide Synthase

**Figure 1:**
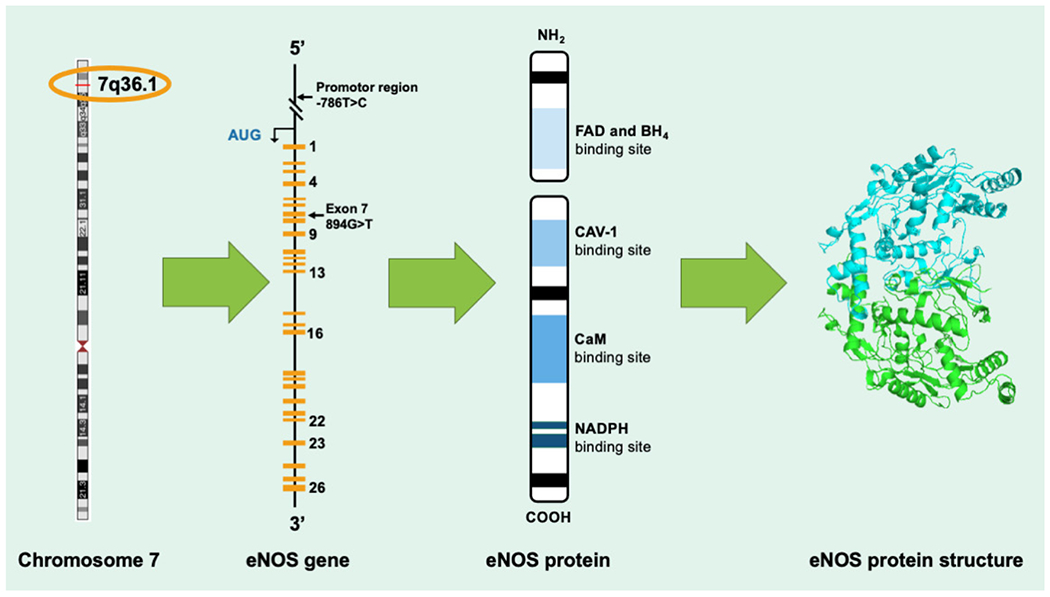
Scheme of the gene encoding endothelial nitric oxide synthase (eNOS). The human eNOS gene is located at 7q35-36 and it contains 26 exons with the start transcription site designated by AUG in the promoter region; polymorphisms are indicated in the promotor region and exon 7. Scheme of the eNOS protein is also shown on the extreme right.

Endothelial NOS or eNOS is encoded by the NOS3 gene that is in the 7q35-7q36 region of chromosome 7 in human beings (Figure 1). The eNOS enzyme protects the CVS, and this is because of its ability to produce one of the most important neurotransmitters/neuromodulators in a proportionate amount that is physiologically beneficial to the CVS.

NO is widely known as an autocrine and paracrine signaling factor that exerts numerous pleiotropic functions, such as modulation of blood flow and circulation, thrombosis, inflammation, immune regulation, as well as neural activity. In addition, a review has summarized the important functions of NO that is produced by eNOS which are its anti-thrombotic and anti-embolic effects, particularly because of NO diffusion across the platelets’ membranes, activating sGC, and ultimately resulting in attenuating platelet aggregation or platelet clumping [8]. Further research in the future will most definitely provide additional important functions of the eNOS protein in the protection of not only the CVS, but also in neuroprotection (see below) of the nervous system as well as other systems, including immunological responses in humans. For example, a review discusses the amount of NO that is generated by eNOS having excellent protective antioxidant properties because of its ability to decrease the formation of superoxide anion, commonly referred to as “free radicals”, mediated via NO-induced increases in the expression of superoxide dismutase, an antioxidant enzyme that catalyzes the conversion of superoxide anion into hydrogen peroxide (H_2_O_2_) [9]. The antioxidant properties of NO produced by eNOS are also due to the up-regulation of heme-oxygenase-I and ferritin expression, that in turn, attenuate superoxide anion concentrations within the vasculature in general [10]. For example, superoxide anion radical (O_2_^∙^) is generated via numerous enzymatic processes, autooxidation reactions, and nonenzymatic electron transfer reactions and produces the most important widespread reactive oxygen species (ROS) [11,12]. In addition, a reaction between O_2_∙ and NO produces a toxic molecule known as peroxynitrite (OONO^−^) that interacts with carbon dioxide (CO2) and changes into highly reactive nitroso peroxocarboxylate (ONOOCO_2_^−^) or peroxynitrous acid (ONOOH). This reaction can oxidize lipids, oxidize methionine and tyrosine residues in proteins, and it can further oxidize DNA to form nitroguanine [13]. Finally, NO can react with other radicals such as H_2_O_2_ and HOCl that are converted into N_2_O_3_, NO_2_^−^ and NO_3_^−^ [14].

### Polymorphisms in eNOS Gene

The eNOS gene is located on chromosome 7q35-36 with well-defined polymorphisms (Figure 1) [15]. This location of eNOS on chromosome 7 has been shown to be highly correlated to several life-threatening cardiovascular disease processes, including ruptures of aneurysms and congenital cardiac anomalies [15].

The polymorphisms in the eNOS gene are closely associated with a higher incidence of cardiovascular diseases (CVD). For example, the C allele for the NOS3-786T > C polymorphism of the eNOS gene causes decreased eNOS expression and subsequently an attenuated production of NO [16]. Patients with such a condition will have an increased risk of developing hypertension [17], preeclampsia [18], diabetic nephropathy [19], retinopathy [19], and migraine [20]. The 786T > C is also associated with higher episodes of coronary vasospasm [21]. In patients with a four-repeat allele of the variable number tandem repeats in this genetic variant, there has been an increased risk of aneurysm ruptures, while the C-allele of the 786T > C single-nucleotide polymorphism variant is significantly associated with the occurrence of aneurysm rupture followed by subarachnoid hemorrhage and vasospasm [22–24].

Another variation known as 894G > T is linked to coronary and carotid artery diseases such as myocardial infarction (MI) and carotid arteriosclerosis [25–27]. The 894T > G eNOS single nucleotide polymorphism has been suggested to be responsible for not only a reduced synthesis of NO but also in the development of essential hypertension, particularly in Caucasians and in North African populations [28]. In addition, a previous publication has shown for the first time a highly significant association of ambulatory blood pressure with eNOS haplotypes in a significant white population living in northern Belgium, further suggesting a direct relationship of eNOS polymorphisms on blood pressure [29]. It appears that the above polymorphisms in the eNOS gene have serious pathological impact on the function of the eNOS gene and are involved with several CVD [30]. Overall, there have been overwhelming cases that the eNOS gene is associated in patients with not only CVD but also subjects with diabetes and congenital heart defects.

### eNOS Dimerization and Uncoupling

**Figure 2:**
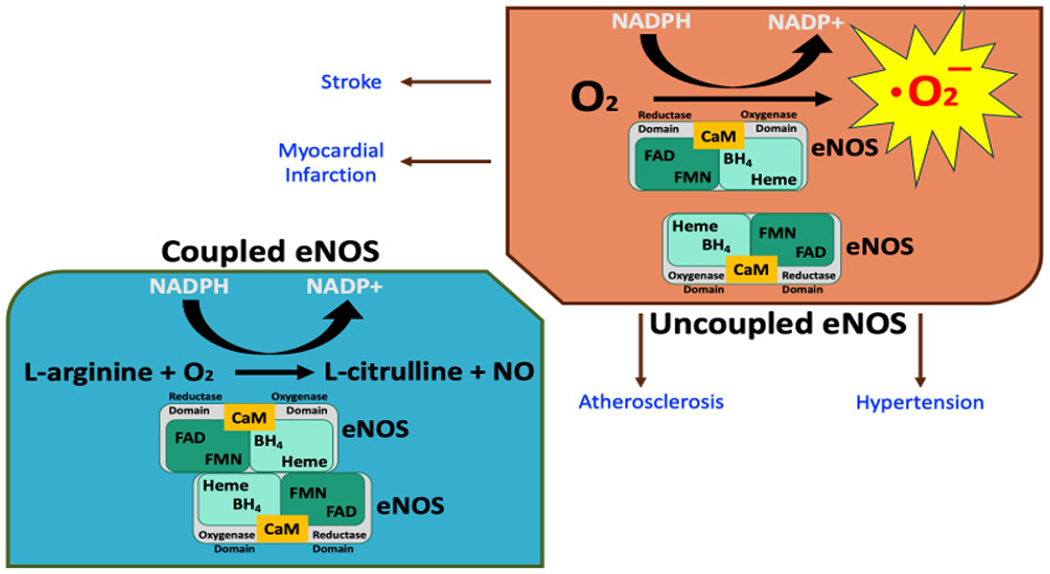
Schematic diagram of coupled versus uncoupled endothelial nitric oxide synthase or eNOS showing that uncoupled eNOS can generate a highly toxic super radical as shown by the abbreviation O2-, the ion superoxide. Other abbreviations: O2 - Oxygen; NADPH - Nicotinamide adenine dinucleotide phosphate hydroxide; NADP+ - Nicotinamide adenine dinucleotide phosphate; FAD - Flavin adenine dinucleotide; FMN - Flavin mononucleotide; CaM - Calmodulin.

Serious clinical and pathological consequences related to not only CVD like atherosclerosis and hypertension but also situations such as diabetes mellitus and nicotine tolerance can occur if the eNOS enzyme dimer gets uncoupled. To understand the consequences of eNOS uncoupling, the structure and function of eNOS as a dimer are briefly discussed. Endothelial NOS is a dimer consisting of two identical monomers that have molecular weights of 134 kD [31]. One of the monomers has a reductase domain that consists of binding sites for nicotinamide adenine dinucleotide phosphate (NADPH), flavin adenine dinucleotide (FAD), and flavin mononucleotide (FMN). The other monomer is an oxygenase domain and consists of heme, zinc, the cofactor tetrahydrobiopterin (BH_4_), and L-arginine binding sites. A calmodulin-binding sequence links the reductase domain with the oxygenase domain (Figure 2). It is now known how NO is synthesized by eNOS from L-arginine and molecular oxygen in the vascular endothelium. Briefly, located at the reductase domain of eNOS, the FMN and FAD molecules shuttle electrons from NADPH to the heme center in the oxygenase domain for this NO production. Heme is reduced by the reductase domain initially, which then binds with O_2_ and forms an oxyferrous complex and reduction of this oxyferrous complex uses electrons derived from NADPH located within the reductase domain. However, interdomain electron transfer from NADPH of the reductase domain to the oxygenase domain appears to be slow. Therefore, eNOS uses BH_4_ cofactor as an electron donor to rapidly reduce the oxyferrous complex, which can dissociate to ferric heme and superoxide in BH_4_-depleted condition [32–34]. In addition, this BH_4_ has been shown to play a role of “redox switch” which means that it can attenuate superoxide release and promote the formation of NO; and that an addition of 7,8-BH_2_ or sepiapterin enhances superoxide release while inhibiting the production of NO [35,36]. Thus, without this BH_4_ cofactor, eNOS shifts to produce O_2_∙-, a dangerous cytotoxic superoxide anion instead of NO; this clinically deleterious phenomenon is known as eNOS uncoupling [37–39]. It should be noted here that this superoxide anion can be generated by both the heme in the oxygenase domain and the flavins in the reductase domain, although FMN has shown to play an insignificant role in eNOS uncoupling. In addition, eNOS uncoupling can also be caused by heme directed inhibitors in addition to an accumulation of endogenous eNOS inhibitors such as asymmetrical dimethylarginine (ADMA) or eNOS S-glutathionylation [39]. The consequences of having such a production of cytotoxic reactive free radical via uncoupled eNOS can be dangerous to the CVS.

An intricate imbalance between the formation of highly cytotoxic ROS and detoxification by low molecular weight antioxidants can be a hallmark for most CVD. For example, oxidative stress induced by eNOS uncoupling plays a notable role in the pathogenesis of arterial hypertension, which is a highly prevalent cardiovascular risk factor of morbidity and mortality in developed countries. The activation and/or overexpression of eNOS allow vasodilation and regulation of systemic blood pressure [40]. Administration of heparin-binding superoxide dismutase, a metalloenzyme that catalyzes the dismutation of superoxide anion into molecular oxygen and H_2_O_2_, which ultimately neutralizes the ROS and in turn, significantly lowers arterial blood pressure in spontaneously hypertensive rats [41]. Conversely, mice with a nonfunctional eNOS fail to induce vascular endothelium-dependent relaxation [40]. As a result, eNOS uncoupling is observed in isolated blood vessels from animals with hypertension. Furthermore, vascular NADPH oxidase is a major source of superoxide in angiotensin-II (AngII) treated hypertensive mice [41]. Therefore, therapeutic interventions that would attenuate or prevent eNOS uncoupling to normalize endothelial function and improve cardiovascular function, in general, have recently become a great interest in pharmacological research.

In addition, eNOS uncoupling may have a significant contribution to the development of atherosclerosis. The vascular endothelial cells have a critical role in not only the regulation of vascular tone but also alterations in platelet function and aggregation, leukocyte adhesion, plaque formation, development of atherosclerosis with plaque, and ultimately the formation of a thrombus. For example, in hypercholesterolemic apolipoprotein E knockout (ApoE-KO) mice, atherosclerosis development accelerates without a proper functioning eNOS [42], further suggesting that eNOS has a protective role on the CVS. Endothelial dysfunction as a result of eNOS uncoupling not only in coronary arteries but also within other peripheral blood vessels as measured by acetylcholine-dependent dilation has been shown to be an early predictor of CVD processes leading to atherosclerosis [43]. Furthermore, eNOS dimerization and uncoupling are also involved in the pathophysiology of arterial stiffness [44] and patients with diabetic retinopathy [45]. Chronic low-grade inflammation within the vascular endothelial cells and the development of endothelial dysfunction with subsequent cardiovascular maladies are closely related to oxidative stress mediated via uncoupled eNOS enzyme [46]. In addition, immune cells (white blood cells or WBC) can express high concentrations of functional NADPH oxidases that can generate elevated levels of ROS resulting in the induction of vascular oxidative stress [47]. Overall, the above mechanisms that lead to oxidative stress vastly contribute to the low-grade inflammation, particularly in the vasculature of the older population [48]. The eNOS uncoupling undoubtedly gives rise to oxidative stress, which has a negative impact on the CVS [49,50], and is implicated in the pathogenesis of hypertension, atherogenesis, and endothelial dysfunction.

### eNOS Regulation

The expression and activity of the eNOS enzyme are carefully controlled or regulated via numerous inter-related mechanistic concepts occurring at the transcriptional, post-transcriptional, and post-translational levels, in addition to its modulation by several physiologic factors that in turn, have the potential to counteract many of the age-related and/or lifestyle-related diseases including cardiovascular and metabolic health. For example, oral supplementation with L-citrulline that can be purchased over the counter from any drug store or online, has been shown to lower blood pressure in both resting conditions in adult humans, and can also lower blood pressure during stress-induced pressor effects [51]. This has been corroborated using animal studies that L-citrulline can protect against the development of atherogenesis in the endothelial cells of the blood vessel linings. In addition, a previous study discusses whether L-citrulline reduces blood pressure (aortic and brachial) following dietary L-citrulline supplementation [52]. It has also been debated if oral administration of L-citrulline effectively reduces blood pressure by approximately 7 mmHg via increasing the production of NO [53]. Furthermore, a most recent meta-analysis of clinical trials shows that L-citrulline supplementation reduces systolic blood pressure while there has been a significant decrease in diastolic blood pressure in studies that uses doses of ≥ 6 g/day [54]. Finally, it is postulated that oral L-citrulline supplementation can enhance exercise performance and recovery [55,56].

It is now known that the transcription factor for eNOS called Sp1 which is also known as specificity protein 1, is a protein that in humans is encoded by the SP1 gene and is critically involved in various cellular processes that may include cell differentiation, cell proliferation, apoptosis, immune responses, chromatin remodeling, and even DNA damage [57]. There are also post-translational modifications such as phosphorylation, acetylation, O-GlcNAcylation, or proteolytic processing that can, in turn, considerably influence the activity of Sp1 via its modulation as an activator or a repressor protein. Besides Sp1, there are other factors that regulate transcription such as binding of factors like Sp3, Ets-1, Elf-1, and also the YY1 to the eNOS promoter region and followed by subsequent methylation of the DNA; this is a critical mechanism of the eNOS transcriptional regulation [58]. There are numerous regulatory factors such as H_2_O_2_ and more than 150 potential transcription factor binding sites for eNOS, such as eNOS-dependent inhibition of the NF-κB subunit p65, that have been shown to produce neuroprotective effects [59,60]. In addition, eNOS is regulated post-transcriptionally via several mechanisms such as modifications of the primary transcript, stability of the mRNA, cellular or subcellular localization, and the transport from the nucleus to the cytoplasm [61]. Finally, modifications of eNOS during its post-translational level are acylation of fatty acid, protein-protein interactions, availability of the substrate and co-factors, and phosphorylation. Furthermore, eNOS enzyme is inactivated because of the strong and direct binding or interaction with caveolin-1 [62]. Lastly but not least, activation of eNOS is dynamically regulated and modulated by multiple phosphorylation sites at tyrosine, serine, and threonine residues [63]. Overall, it is now well-established that the expression/activity of the eNOS isoform can be regulated by several important mechanisms.

If the bioavailability of NO as generated by the eNOS enzyme is decreased there may be an increased risk of the development of age-, environmental-, dietary-, and lifestyle-related diseases such as hypertension, atherosclerosis, insulin resistance or type 2 diabetes, and other CVD [64]. Therefore, it appears that eNOS is a crucial NOS isoform that regulates and modulates the function of vasculature and the function of the CVS [65]. A thorough review of the literature shows that the expression and activity of eNOS and thus the downstream production of NO are initiated or modulated by numerous stimuli, neurotransmitters, and even hormones that are mediated via Ca^2+^-dependent or Ca^2+^-independent mechanisms. For example, eNOS can be activated by shear mechanical stress due to the flow of blood (laminar and turbulent) in a PKA-dependent manner, bradykinin, histamine, and 17β-estradiol or the major female sex hormone, and many other stimuli [66–72]. The mechanism of ACh-, bradykinin-, and histamine-induced modulation of the eNOS activity is that they act on their respective specific receptors on the endothelial cell membrane and increase the intracellular concentration of Ca^2+^, which binds to calmodulin, and finally activates the calmodulin-binding domain of eNOS. This ultimately facilitates the electron flux from the reductase to the oxygenase domains of eNOS and produces NO. Furthermore, post-translational modification via phosphorylation of the eNOS enzyme is also equally critical for activation of the enzyme as it enhances the active influx of electrons from the reductase to the oxygenase domains in order to produce NO [73]. In addition to the above mechanisms, protein kinase A (PKA) and protein kinase B (Akt) activate eNOS by phosphorylating Ser1177 in response to various stimuli and on the other hand, H_2_O_2_ and bradykinin will activate eNOS via increasing Ser1177 phosphorylation and Thr495 dephosphorylation in order to produce NO [74–76]. In addition, the levels of BH4 are significantly reduced in the plasma of spontaneously hypertensive rats when compared to the WKY rat breed in the aorta of insulin-resistant rats, and in deoxycorticosterone acetate (DOCA)-induced-salt-treated hypertensive rats. A reduced eNOS expression can decrease the production of NO as observed in adult spontaneously hypertensive rats, however, other studies also show that endothelial dysfunction is associated with an elevated rather than an attenuated eNOS expression [77].

In addition to the above mechanisms, several pharmacological agents can modulate the expression of eNOS. Angiogenesis or formation of new blood vessels is involved in the growth of tumors where both vascular endothelial growth factor (VEGF) and NO are interchangeably involved. Aspirin, an over-the-counter medicine that may be orally administered to prevent heart attack and stroke, has been shown to inhibit eNOS [78]. Aspirin taken at bedtime significantly diminishes plasma renin activity and excretion of cortisol, dopamine, and norepinephrine in 24-hour urine that, in turn, may reduce blood pressure [79]. However, there is also a discrepancy in the literature about whether older publications overstate the benefits of asprin [80]. Furthermore, angiogenesis that is induced by the eNOS gene has been shown to be mediated via the expression of VEGF in a rat model of hindlimb ischemia [81]. In addition, NO production and regulation of eNOS phosphorylation are modulated via treatment with troglitazone, an activator of peroxisome proliferator-activated receptors (PPARs) suggesting the involvement of PPAR-gamma-dependent and PPAR-gamma-independent signaling pathways [82]. Therapeutic targeting of up- and down-stream in the cascade of the eNOS/NO/sGC/cGMP axis has been a recent focus of scientists aimed at the treatment of several CVD (See Daiber *et al.,* 2019 for a review [41]). Agonists and antagonists of the sGC enzyme can be a future novel class of “repair” medications that can modify the oxidatively damaged sGC enzyme, and thereby can be prescribed for the treatment of not only pulmonary hypertension or heart failure, but also in other situations such as hypertension, erectile dysfunction, atherosclerosis, restenosis, thrombosis, and inflammation [83]. Sepiapterin or 2-amino-6-[(2S)-2-hydroxypropanoyl]-7,8-dihydro-1H-pteridin-4-one is a member of the pteridine class of organic chemicals and is metabolized into BH4 via a salvage pathway that improves endothelial function by preventing eNOS uncoupling in vascular tissues *in vivo* [84]. Endothelial dysfunction in spontaneously hypertensive rats is particularly based on vascular remodeling that cannot be treated with antihypertensive medications. Therapeutic treatment with the AngII subtype AT1-receptor-blockers, statins, and organic nitrates that have antioxidant effects, significantly improves the expression of GCH-1 or GTP cyclohydrolase I enzyme which is a part of the folate and biopterin biosynthesis pathway that can normalize BH_4_ levels, revitalize eNOS, and improve endothelial function in pathological states such as diabetes, hypertension, atherosclerosis, and nitrate tolerance [85,86]. AT1-receptor blocker valsartan induces NO production via Src/PI3K/Akt-dependent phosphorylation of eNOS, demonstrating vasoprotective AT-1 receptors in upregulating NO production [87]. These data are supported by most recent studies suggesting that uninhibited AT1-receptors can decrease eNOS activity [88]. Overall, AT1-receptor participates in eNOS function, which in turn regulates blood vessel homeostasis.

Hypercholesterolemic patients have impairment in the endothelial function [89]. Because of its rigid ring structure, cholesterol is an important component that dictates the fluidity of the cell membrane. As cholesterol content in the cell membrane increases, the flexibility of the plasma membrane decreases resulting in altering the cascade of signaling pathways [90]. As a result, cholesterol-induced impairment of NO production can therefore be modulated by various factors including caveolin, AMPK, and NF-kB [91,92]. Hypercholesterolemic patients carrying the CC genotype for the g.-786T > C polymorphism when compared to TT carriers receiving a protocol of statin therapy have more NO bioavailability [93]. Furthermore, in both groups of hypertensive patients carrying the TC/CC genotypes and the C allele for the g.-786T > C polymorphism there are comparatively effective antihypertensive responses with the administration of the ACEi enalapril [94]. Also, male subjects with symptoms of erectile dysfunction who carry the C allele for g.-786T > C eNOS polymorphism respond much better when treated with sildenafil, the PDE-5 inhibitor [95]. The above reports show that pharmacological agents such as statins, ACEi, and PDE-5 inhibitors may have the ability to improve or resolve an impaired production of NO in human subjects who carry the variant allele/genotype for g.-786T > C eNOS polymorphism and indirectly lessen the susceptibility of CVD.

## Endothelial Nitric Oxide Synthase and the Cardiovascular System

The previous sections discuss the role of eNOS in controlling a healthy CVS and that a dysfunction of the eNOS enzyme is correlated with a diverse pathogenesis of CVD. In the following sections, the roles of eNOS in cardiac myocytes, heart functions, vasculature, different regions of the brain, cardiovascular reflexes, and cardiovascular regulatory pathways involving the autonomic nervous system (ANS) will be elaborated.

### eNOS in Cardiac Myocytes

The amount of NO as generated by eNOS has four mechanistically different effects on the myocardial cells, such as (1) eNOS attenuates the strength and frequency of cardiomyocyte contractility; (2) eNOS enhances cardiac myocyte relaxation by increasing its distensibility; (3) eNOS inhibits mitochondrial respiration; and (4) eNOS improves the efficacy of myocardial oxygen consumption. Overall, eNOS protein can partially inhibit cardiac myocyte functions which may be eventually cardioprotective in both physiological and pathological states. Most of the previous research on eNOS and cardiac myocytes are focused on myocardial eNOS involvement in the muscarinic regulation of inotropy; eNOS and caveolin interaction in cardiac myocytes; role of PDE-5 inhibitors on eNOS and myocytes; ROS and eNOS-dependent trafficking of angiotensin-II (AngII) type 2 receptors (AT2R) in cardiac myocytes; role of Ca^2+^ on eNOS activation; the interaction between purinergic P2X4 receptors (P2X4R), a ligand-gated ion channel, with the cardioprotective Ca^2+^-dependent eNOS enzyme; and how myocyte-specific overexpression of eNOS protects myocardial ischemia/reperfusion injury; among others.

The eNOS-induced NO production plays a critical role in the regulation of coronary vasomotor tone, inhibition of platelet aggregation, and prevention of cell adhesion to the vascular endothelium under normal physiological conditions within the CVS, in addition to modulation of myocardial contractile function *in vitro* and *in vivo* [96]. However, in pathological states like myocardial ischemia, there is a significant impairment of endothelium-dependent coronary relaxation, altered myocardial contractility, and the potential risk of life-threatening cardiac arrhythmias. A previous report studies the role of eNOS in MI followed by reperfusion injury by subjecting wild-type and eNOS-deficient mice to a 20-minute coronary artery occlusion followed by 120 minutes of reperfusion, and indeed, eNOS gene deletion has been shown to aggravate ischemic and reperfusion injury [97]. Another study demonstrates that upregulation of myocyte-specific NO-cGMP generation provides significant cardio-protection against MI and reperfusion injury due to blunted myofilament sensitivity to Ca^2+^ and subsequent improvement of cardio-mechanical activity [98]. With respect to the involvement of eNOS in the muscarinic regulation of negative inotropy of the heart, eNOS overexpression in left ventricular (LV) myocytes has been shown to enhance the negative inotropic effects of muscarinic receptor stimulation both *in vivo* and in cultured neonatal myocytes99. A previous investigation also reveals that myocardial eNOS is involved in both the β-adrenergic-mediated positive inotropy and muscarinic regulation of negative inotropy [99]. Another study also demonstrates that muscarinic type-2 cholinergic receptors (M2AChR) signaling in cardiac myocytes is targeted by the involvement of M2AchR with sarcolemma caveolae and eNOS activation [99].

The interaction between eNOS and caveolin within cardiac myocytes is also well-documented by the fact that eNOS is targeted to plasmalemmal caveolae within endothelial cells. Caveolae are specialized domains of the plasma membrane that host sequester signaling proteins such as transmembrane proteins known as the caveolins. In cardiac myocyte lysates, nearly all the eNOS is immunoprecipitated by antibodies to caveolin-3 and, conversely, by the eNOS antiserum immunoprecipitated caveolin-3. These studies establish expression of eNOS in cardiac myocyte caveolae and document tissue-specific and quantitative associations of eNOS with caveolin [100]. Another study shows the cardiac myocyte-protective effects of microRNA-22, an anti-apoptotic agent, during MI and reperfusion are mediated via disrupting the caveolin-3/eNOS signaling [101]. On the other hand, the role of PDE-5 inhibitors on eNOS and myocytes is also studied extensively, particularly following the FDA approval of sildenafil. A previous report using reverse transcription-PCR, western blots, and immunohistochemical assay confirms the expression of PDE5 within mouse cardiomyocytes and that sildenafil causes overexpression of mRNA and protein contents of eNOS in the myocytes [102]. This sildenafil-induced protection against necrosis and apoptosis is, however, reduced within the myocytes of eNOS knock-out mice. In addition, the cytosolic Ca^2+^-induced apoptosis within rat cardiomyocytes is mediated via mitochondrial eNOS/NO/cGMP/protein kinase G pathways [103]. In this paragraph the interactions among eNOS, caveolin, and PDE-5 inhibitors within the cardiac myocytes are highlighted.

A previous study demonstrates that pre-treatment of LV myocardial cells with AT1R or AT2R antagonists and ROS scavengers such as apocynin inhibits AngII-upregulation of the nNOS protein, and that AngII elevates the eNOS-Ser1177 while attenuating eNOS-Thr495 suggesting a concomitant activation of eNOS [104]. Another study demonstrates in a genetic mouse model that AT2Rs produce anti-hypertrophic results in cardiac remodeling following a model of myocardial cryoinjury and the suggested link is an over-expression of cardiac eNOS protein to AT2R activation [105]. The role of Ca^2+^ on eNOS activation has also been extensively investigated. Both NO and H_2_O_2_ play critical roles in physiological and pathological states within the cardiac myocytes, however, why or how the H_2_O_2_-modulated phosphorylation pathways regulate eNOS in these cells is still incompletely understood. Early studies document that H_2_O_2_ promotes Akt phosphorylation that is dependent upon activation of the L-type Ca^2+^-channels and suggest that Ca^2+^- and PKC-dependent signaling pathways are involved in the modulation of cardiac myocyte eNOS activation by H_2_O_2_ [106], thus further emphasizing the protective action of eNOS on the CVS. On the other hand, endothelial NO production by eNOS requires both Ca^2+^ signaling and Akt phosphorylation and can be modulated by inhibiting or promoting either one of the pathways. The activation of eNOS and subsequent NO production in endothelial cells can be inhibited by mechanical sensors within the myocytes involved in the NO production reaction cascade which is relevant to heart diseases with significant clinical applications [106]. Finally, there is an interaction between P2X4 receptors (P2X4R) which is a ligand-gated ion channel and the cardioprotective Ca^2+^-dependent eNOS effects. The novel association between P2X4R and eNOS suggests that cardiac-specific activation of eNOS is more cardioprotective than an increased bioavailability of systemic eNOS [107]. In the CVS, in addition to P2X4R, P2X7 plays major roles in inflammation, cellular metabolism, apoptosis, and cellular death; and thereby posing a serious concern in the development of MI, stroke, and vascular diseases such as atherosclerosis, hypertension, and thromboembolism [108].

### eNOS, the Heart, and Cardiac Functions

Experiments with isoform-specific inhibitors or genetic deletion experiments have discovered specific regulatory/modulatory roles for each of the three NOS isoforms that are subserved by their subcellular localization and signaling pathways in the cardiac muscle and the vasculature. Moreover, using immunohistochemistry, western blot analysis, and NOS catalytic assay, a previous study demonstrates the expression and localization of eNOS in the myocardium and using eNOS mutant (eNOS−/−) mice as an animal model to examine the role of eNOS in cardiac function [109]. It is now well established that the eNOS enzyme plays a critical physiological role in regulating and modulating cardiac functions.

The nNOS, eNOS, and iNOS isoforms are located within the sarcolemmal caveolae, sarcoplasmic reticulum (SR), T-tubular junctions, and the mitochondria within a cardiac myocyte. The effects of the eNOS on cardiac functions including myocardial contractility vary according to different stimuli such as myocardial stretch due to increased blood volume or LV hypertrophy, oxidative stress, β_2_/β_3_ adrenergic and/or M2 muscarinic receptor stimulation that occur via the production of NO [110]. The anti-adrenergic inotropic effects following β_3_ adrenoceptor-dependent activation of eNOS and the production of NO are mediated via cGMP-dependent modulation of β-adrenergic cAMP-protein kinase A (PKA) pathways mediated by L-type Ca^2+^ channels, ryanodine receptor Ca^2+^-release channel type 2 (RyR2), phospholamban, and the cardiac enzymes, troponin I (TnI) and troponin C (TnC) [110]. In contrast, NO produced by eNOS elicits positive inotropy, particularly following myocardial stretch mediated via activation of protein kinase B (PKB), nitrosylation of RyR2 and L-type Ca^2+^ channels, activation of adenylate cyclase, and RyR2 through phosphorylation of protein kinase G (PKG). In hypertensive models of eNOS−/− mice, there are reports of reduced arteriolar density, reduced Ca^2+^ within the SR, and increased expression of K+-channels in ventricular myocytes [111,112]. On the other hand, very high levels of eNOS expression/activity and overproduction of NO in transgenic mice that have overexpression of the eNOS gene show significantly attenuated basal myocardial contractility that is most probably mediated via desensitization of cardiac myofilaments to Ca^2+^. Overall, the eNOS enzyme has been associated with both positive and negative inotropic effects on cardiac functions. It should be mentioned here that the cardioprotective and useful effects of physical exercise following an attack of MI are crucially dependent on the expression of endogenous eNOS within the heart and that the lack of one of the eNOS alleles can nullify all the beneficial effects of regular exercise [113].

The cardiac eNOS-derived NO and the endothelin-1 (ET-1) systems are closely related, and both play a crucial regulatory role in the modulation of cardiac physiology: An imbalance in these two systems can be deleterious in eliciting several CVD such as hypertension and stroke. One report investigates the cardiac effects in mice models of excessive expression of ET-1 along with lowered expression of eNOS while comparing LV hemodynamics and cardiac morphology. The mouse models include wild-type mice, ET-1 transgenic (ET+/+), eNOS knockout (eNOS−/−), and a model of ET+/+ along with eNOS−/− mice. The results suggest that overexpression of cardiac ET-1 along with simultaneous under-expression of eNOS modulate regulatory proteins and signaling cascades that in turn, affect oxidative stress, myocardial strength and contractility, and energy metabolism [114,115]. The uncoupling of eNOS that results in a significant reduction of NO production will have a potentiated elevation in myocardial superoxide. The resultant effects are compromised coronary artery vasodilatation, elevated amounts of myocardial collagen with decreased capillary/myocyte ratio, stiffening of the heart, and end-diastolic LV stiffness [116]. It should be mentioned here that an episode of acute MI happens in mice that lack all the three NOS enzymes [117]. Astragaloside IV (AST, C41H68O14) is a small molecular saponin and the major active component extracted from Astragalus membranaceus (Fisch), a plant particularly valued in traditional Chinese medicine. It has been shown to have anti-hypertensive, positive inotropic, anti-inflammatory, anti-oxidative activities, and may block the hypertrophy and apoptosis of myocardial cells following heart failure [118]. This extract has been shown to reverse oxidative stress-mediated injury, repair NO-signaling cascade, and improve myocardial LV diastolic dysfunction in a rat model of metabolic syndrome mediated via activation of the eNOS/NO/cGMP pathways [118]. On the other hand, in humans, it has been reported that three eNOS polymorphisms i.e., T-786C, VNTR4a/b, and Glu298Asp, and their haplotypes have a better prognosis on the morbidity and clinical outcomes in heart failure patients with systolic dysfunction [119]. HSPA12B, an endothelial-cell-specifically expressed heat shock protein, has been shown to reduce cardiac systolic dysfunction and remodeling following an episode of MI in transgenic mice that is mediated through an eNOS-dependent mechanism [120]. Finally, it has been shown that the anti-hypertensive drug olmesartan (AT1R antagonist) improves cardiac systolic dysfunction associated with significant cardiac remodeling of Dahl salt-sensitive hypertensive rats with end-stage congestive cardiac failure mediated via an Akt/eNOS pathway [121]. In summary, the role of eNOS on cardiac functions is well-established and that an impaired eNOS-derived NO production exerts profound deleterious effects on several CVD such as hypertension, stroke, and MI.

### eNOS and the Vasculature

**Figure 3:**
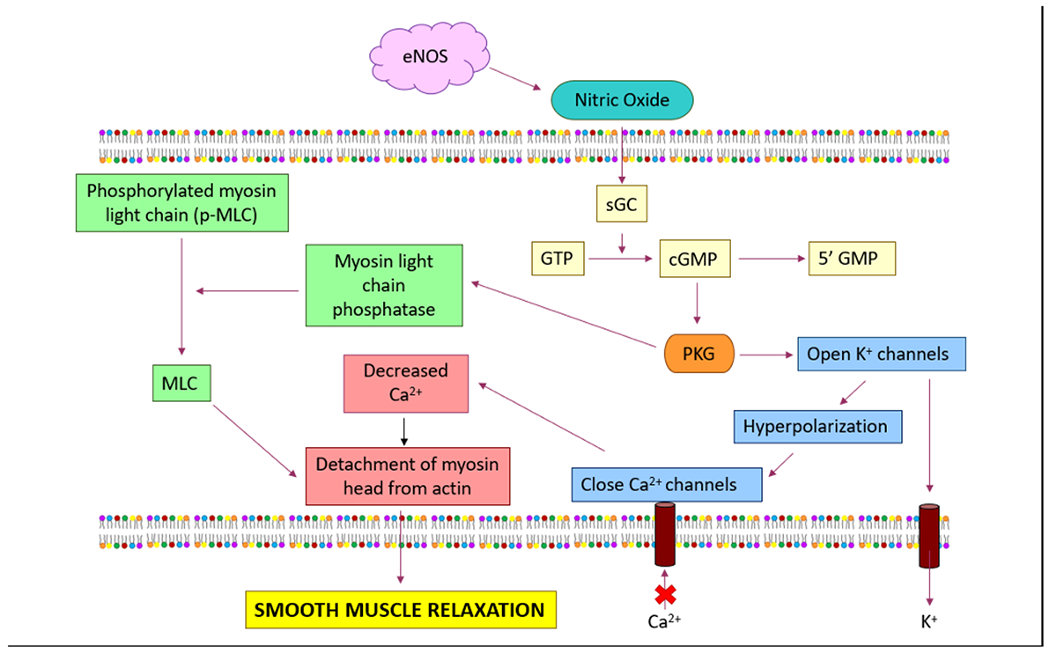
A schematic diagram of how eNOS via NO causes smooth muscle relaxation in vascular smooth muscle cells. Abbreviations: cGMP – cyclic guanosine monophosphate, GTP – guanosine triphosphate, sGC – soluble guanylate cyclase, PKG – cGMP dependent protein kinase.

One of the most important functions of the eNOS enzyme is to elicit the production of a sufficient amount of NO that regulates, modulates, controls, and maintains the vascular tone of the CVS. Diffusion of NO across the vascular smooth muscle cell membranes activates the enzyme soluble guanylate cyclase (sGC) and catalyzes the conversion of guanosine triphosphate into cyclic guanosine monophosphate (cGMP) [122]. This cGMP is responsible for activating protein kinase G (PKG) and eliciting phosphorylation of cellular targets via reducing intracellular Ca^2+^ concentrations. The ultimate result is vascular relaxation or vasodilation (Figure 3).

Recent advances in technology as well as imaging engineering, such as contrast enhanced images for vascular phenotyping to analyze defects in vascular perfusion/structure are emerging. For example, the vascular tree of humans is a three-dimensional organization and hence, two-dimensional assessments of the vascular architecture do not provide enough information, particularly about vessel thickness, volumetric abundance, and connectivity. In addition, any histomorphometric analysis is also shown to be destructive and much time consuming. Furthermore, researchers have developed techniques using x-ray micro-computed tomography (microCT) that will generate a three-dimensional (3-D) vision of microscopic structures based on their attenuation of x-rays [123]. Thereafter, modern polychromatic 3-D microCT systems are invented that are capable of generating image volumes as small as 1 μm with a meager 3-to-5-hour scan acquisition time. Nowadays, contrast enhanced 3-D microCT has become a powerful mechanistic tool for an enhanced telescopic visualization of any vasculature.

Using the 3-D x-ray microCT, investigators have shown that renal cortical arteries of the kidneys in eNOS−/− mice have severe defects in vascular perfusion and morphological structure [123]. Another study also has quantified changes in eNOS mRNA levels throughout the canine vasculature using competitive PCR assay [124], and that the ACh- or bradykinin-induced vasodilation of forearm resistance vessels in humans is mediated via the eNOS protein [125]. Indeed, exercise training increases the amount of eNOS in the coronary arterial microcirculation [126]. In addition, estrogen increases NO release in human coronary arterial endothelial cells and is mediated by the upregulation of eNOS [127]. Regular exercise and physical activity in patients with coronary ischemic disease improve the function of endothelial cells via increasing the phosphorylation of the eNOS protein. In addition, regular exercise training elicits an increased eNOS coupling that in turn, restores relaxation of coronary arteries of heart failure rats [128]. It has been shown that C-reactive protein (CRP), the prototypic marker of inflammation, decreases eNOS expression along with its bioactive functions in human aortic endothelial cells [129]. It has been widely known that oxidative stress can lead to endothelial dysfunction and several CVD risk factors that cause serious morbidity. Particularly, as mentioned previously, endothelial function is dependent upon eNOS and that a properly functioning eNOS activity within the endothelium is critical for vascular integrity and homeostasis. Moreover, exposures to loud noise, mental stress, and environmental factors represent a strong trigger for oxidative stress that may ultimately lead to atherosclerosis and endothelial dysfunction [41]. Thus, there has been a new focus of therapeutic implications on eNOS function or dysfunction in CVD including the importance of regular vitamin C intake and/or selective antioxidant medications such as xanthine oxidase inhibitors [41].

In addition to the coronary arteries and the aorta, previous studies have shown *in vivo* adventitial expression of recombinant eNOS gene in cerebral arteries [41]. Another study also investigates the kinetics of NO elicited by eNOS following transient global forebrain ischemia in mice that show the eNOS-derived NO production in the striatum region of the brain after reperfusion is closely related to eNOS activity [130]. It is now known that rupture of an aneurysm of any cerebral blood vessel and subsequent subarachnoid hemorrhage can cause fatal results, including death, and Redundant and that one of the mechanisms of cerebral aneurysm is a result of chronic inflammation in endothelial cells of arterial walls due to an imbalanced hemodynamic force. It is known that eNOS provides protection of the arterial wall layers from vascular inflammation mediated via relieving hemodynamic force and through the production of NO. A defective function of the eNOS isoform could be compensated by upregulation of the nNOS enzyme within cerebral arteries and that a complimentary balance between eNOS and nNOS has a beneficial role in cerebral aneurysm [131]. Tea polyphenols are rich in green tea, which accounts for 30%-40% of the dry weight of tea leaves. Tea polyphenols mainly contain four kinds of monomers: epigallocatechin-3-gallate, epicatechin, epigallocatechin, and (-)-epicatechin-gallate. A recent study shows that the epigallocatechin-3-gallate found in tea may reduce neuronal apoptosis in a stroke rat model via PI3K/AKT/eNOS signaling pathways [132]. Furthermore, another study suggests that anticholesterolemic medications such as statins protect against stroke by enhancing clot lysis via multiple mechanisms involving the eNOS enzyme [133]. The widely prescribed anti-hypertensive drug, losartan, an AT1R blocker, can attenuate the cerebral ischemia-reperfusion injury via PI3K/Akt-mediated eNOS phosphorylation [134]. A recent study investigates the role of vitamin D3 in the upregulation of the eNOS protein within the endothelium of cerebral arteries after subarachnoid hemorrhage in rats, and indeed, this vitamin can reduce the cerebral artery remodeling through VDR/AMPK/eNOS dimer phosphorylation pathways [135].

The Ca^2+^ channel and Ca^2+^signaling also play an important role in mediating the eNOS activity. Several transient receptor potential (TRP) cation channels have been shown to regulate eNOS activity and NO production, as well as their role in CVD. TRPP2, also known as polycystin-2, is regulated by fluid-shear stress, and it initiates a signaling pathway cascade of Ca^2+^, calmodulin, Akt/PKB, and PKC [69,70]. Dysfunction in sensing the shear-stress or signaling cascade results in vascular hypertension [66,67]. The TRP subfamily V member 1 (TrpV1) or Subfamily A Member 1 (TRPA1) is also involved in the regulation of eNOS activity. The Ca^2+^ influx triggered by TRPV1 activation in endothelial cells can induce the activity and expression of eNOS [136]. TRPV1 agonism inhibits endothelial cell inflammation via activation of eNOS/NO pathway and inhibits hypertension-induced cellular inflammation in endothelial cells via Ca^2+^/PI3K/Akt/eNOS/NO pathway [137].

The eNOS-derived NO has a critical role in maintaining vascular function, exerting an antithrombotic action, and that an attenuated expression of eNOS is closely related to stroke and Alzheimer’s disease (AD), the most common type of dementia associated with neurovascular dysfunction. A previous study indeed shows that a deficiency of eNOS using a heterozygous eNOS+/− mice to mimic partial deficiency of eNOS, causes spontaneous thrombotic cerebral infarction and signs of cognitive impairment [138]. Overall, it can be summarized that the eNOS protein has a very critical function in vascular homeostasis and that a lack or defective eNOS can lead to serious pathological consequences for developing CVD.

## Endothelial Nitric Oxide Synthase and the Nervous System

One of the multiple actors impinging on the nervous system is NO that plays an extensive role in both the central and peripheral nervous systems. All the three isoforms, (eNOS, nNOS, and iNOS) are intricately involved in both normal physiological and pathological functions of the central nervous system related to the control of the CVS and diseases such as hypertension and stroke.

### eNOS and the Central Nervous System

The glial cells as well as the pericytes surrounding the cerebral blood vessels contain guanylyl cyclase (GC) that is stimulated by eNOS-derived NO release from endothelium. The eNOS protein is abundant in both cerebral vascular endothelial cells and motor neurons; and in normal physiological processes, the eNOS-derived NO regulates blood flow, circulatory changes, and acts as a messenger during long-term potentiation (LTP). On the other hand, in pathological situations such as hypertension and cerebrovascular accidents, the function/expression/activity of the eNOS isoform is impaired that may contribute to a reduction in blood flow, dysfunctional oxygen/metabolites delivery, massive release of Ca^2+^, glutamate, ROS, super radicals, and other toxicological agents from the brain tissues in association with a disturbed blood brain barrier [1,139].

The eNOS isoform via producing NO also regulates the microcirculation within the cerebral vessels, inhibits platelet aggregation, and blocks leukocyte adhesion and migration [140,141]. Under physiological conditions, the concentration of NO molecules fluctuates within a range of low values that are generated by nNOS and eNOS, however, because of its Ca^2+^-independent activation, the iNOS isoform can produce a large amount of NO that is about 100 to 1000 times higher that can turn into a toxic super radical [142,143]. Thus, if a question arises, “Is NO a friend or a foe”, the answer is both. Nitric oxide in moderate amounts can be neuroprotective while NO in large concentrations will be neurotoxic or cytotoxic. Interrupting the gene that encodes eNOS will lead to spontaneous systemic and pulmonary hypertension and will inhibit growth factor (GF)-mediated angiogenesis in experimental mice [144,145]. Even in animal models of inflammatory disease, the disruption of the blood brain barrier is mediated by dysfunctional eNOS activity [146] and that eNOS is also impaired during systemic infection, which may lead to a dysfunctional cerebral microcirculation [147]. In addition to the role of the nNOS isoform on the CVS1, many studies have demonstrated the localization of the eNOS enzyme within different regions of the brain, particularly the nucleus tractus solitarii (NTS), the rostral ventrolateral medulla (RVLM) and the caudal ventrolateral medulla (CVLM), major areas that are closely related to the central regulation of circulation and reflexes such as the baroreflex and the Exercise Pressor Reflex [1,139]. The laboratory of Hirooka *et al.* has developed a novel *in vivo* technique for an effective eNOS gene transfer into the NTS of conscious rats and performed several studies to determine the effects of eNOS within the NTS on the sympathetic autonomic nervous systems (SANS) [148–150]. In addition to the NTS, studies with eNOS and its effect on the RVLM in both acute and anesthetized models have shown both sympatho-excitatory and sympatho-inhibitory responses [148–150]. Overexpression of the eNOS isoform within the RVLM produces a prolonged sympatho-inhibitory effect. Microinjection of bicuculline, a γ-amino butyric acid (GABA) receptor antagonist, into the RVLM increases blood pressure and an overexpression of eNOS in addition to a rise in localized GABA concentration [151].

Numerous mechanisms, neurotransmitters, receptors, and pathways are involved within the RVLM and the CVLM regions of the medulla that regulate the CVS. Overall, the NO as generated from an overexpression of the eNOS protein within the RVLM is known to cause sympathoinhibition via modulating GABA release. Previous research results also corroborate with the above findings where microdialysis of the eNOS blocker, L-N(5)-(1-iminoethyl)-ornithine (L-NIO; 10 μM), into the RVLM in anesthetized rats did not change the resting blood pressure, heart rate (HR), glutamate concentration, and GABA levels; however, a higher 50 μM dose of L-NIO administered into the RVLM increases blood pressure, HR, and localized glutamate levels while decreasing GABA levels [152]. Totally opposing effects are observed when the eNOS antagonist is administered into the CVLM [152]. Overall, it is now known that cardiovascular control mechanisms within the medulla are maintained by a balance between excitatory and inhibitory interactions among eNOS, glutamate, and GABA [139]. Moreover, the central nervous system exerts profound cardiovascular effects via the autonomic nervous system, and the following subsections will focus on the role of eNOS on both the SANS and the parasympathetic autonomic nervous systems (PANS) that regulate and control the CVS.

### eNOS and the Sympathetic Autonomic Nervous System

The SANS and the CVS are intrinsically linked through nerves such as the cardiac sympathetic nerves, various adrenergic receptors such as α and β adrenoceptors, and neurotransmitters or neurohormones such as epinephrine and norepinephrine. In addition, the SANS sends its sympathetic fibers to major blood vessels, adrenal glands, and the kidneys [153]. As a result, the function of SANS is more extensive with both direct and indirect control of most of the cardiac and vascular functions. When activated, the SANS releases norepinephrine that binds to β-adrenergic receptors and activates adenyl cyclase. Through downstream signaling cascade, HR rises due to the increase of diastolic depolarization in the sinoatrial or the sinus atrial (SA) and atrioventricular (AV) nodes, thereby regulating both cardiac rate and stroke volume [153]. Besides its cardiac function, norepinephrine released from the sympathetic axon can also bind to α1- or β2-adrenergic receptors on vascular smooth muscle cells resulting in vasoconstriction or vasodilation, respectively, in addition to maintaining a resting vascular tone [154]. Thus, there is a pivotal role of the SANS in cardiac and vascular functions.

In the brain, NO is known to modulate the activities of the SANS and to regulate cardiovascular activities such as control of mean atrial pressure (MAP) and HR fluctuations [1,139]. In addition, acting within the paraventricular (PVN), supraoptic nuclei (SON) of the hypothalamus, and the posterior hypothalamus, NO has been shown to be an important neurotransmitter regulating the neurohumoral control on the CVS [155]. Along with nNOS, a significant amount of eNOS is expressed in the hypothalamic PVN and SON. Moreover, the basal bioavailability of NO in the PVN is maintained by both the eNOS and nNOS isoforms. However, NO synthesis by eNOS primarily contributes to the tonic NO levels due to its ability to maintain a constant NO bioavailability regardless of cytosolic Ca^2+^ concentrations. Moreover, eNOS-derived NO regulates the neuronal activity of the hypothalamic PVN and modulates sympathoexcitatory effects on the circulation. Reduced NO levels within the PVN can increase sympatho-excitation in rats with heart failure155. Substantial vasoconstriction can also occur if the sympathetic outflow is overactivated153. In addition, hyperactivity of the SANS can lead to arterial hypertension and heart failure and plays a significant role in the pathogenesis of CVD [156]. Before exploring the relationship among NO, the SANS, and CVD such as hypertension or heart failure, the regulatory effect of NO on the SANS via the medullary cardiovascular control regions like the RVLM and the CVLM is discussed in the following sections.

The RVLM is a vasomotor center and is known as the pressor region that regulates the sympathetic outflow from the brain while the CVLM is a depressor region [157]. Through the NTS and the PVN in the hypothalamus, the RVLM receives stimulus from baroreceptors, chemoreceptors, and visceral receptors. An overexpression of eNOS in the RVLM results in an increase in local NO production via modulating the release of GABA and glutamate and can be sustained for several days [158]. To further elaborate, NO activates N-methyl-D-aspartate (NMDA) receptors localized on the pre-sympathetic neurons of the RVLM and triggers the release of GABA. As mentioned above, the production of NO from an overexpression of eNOS in the RVLM exerts a sympathoinhibition effect. In contrast, sympathoexcitation in the RVLM is mediated by oxidative stress that can be produced from the activation of the AT1R/NADPH oxidase within the RVLM. Moreover, oxidative stress in the RVLM can also come from alternative sources such as xanthine oxidase, mitochondria, NADPH oxidase, and uncoupled eNOS. Nonetheless, AT1R antagonists in the RVLM elicit an inhibitory effect on the SANS. Therefore, the most notable source of oxidative stress in RVLM might come from AT1R/NADPH oxidase instead of the other sources as mentioned above [158].

Activation of the SANS increases NO release that can be modulated by altering sympathetic nerve activity that in turn, contributes to an increased blood flow during and after exercise. In both animal and human studies, sympathetic nerve activity can stimulate NO release from eNOS and also from the nNOS protein [159]. This production of NO by eNOS contributes to functional sympatholysis or local attenuation of SANS-mediated vasoconstriction in exercising muscle and/or to direct vasodilation via β2 adrenoceptors in the skeletal blood vessels during exercise. It has been known that NO derived from eNOS regulates blood flow under physiological conditions in humans, however, human studies with a selective nNOS inhibitor, SMTC, demonstrate that the basal vascular tone in forearm and coronary circulatory vasculature are regulated via the nNOS isoform, whereas eNOS is responsible for the vasodilation in response to either pharmacological manipulations or stimulus from shear stress [160,161]. Another study using non-selective NOS inhibition with L-NMMA shows a potentiated vasoconstriction of forearm blood vessels in response to low body negative pressure, suggesting that reflex sympathetic vasoconstriction is opposed by eNOS-derived NO [162]. It is possible that the potentiated vasoconstriction due to sympathetic stimulation is because norepinephrine release in response to low body β as previously described in animal and human studies [159]. Thus, eNOS-derived NO can attenuate β1-adrenergic receptor mediated vasoconstriction. Another study has shown that epinephrine significantly increases eNOS expression or activity in cultured bovine aortic endothelial cells (BAEC) and that this activation is associated with elevated phosphorylation of eNOS at Ser(1179) along with an attenuated eNOS phosphorylation at the inhibitory phosphoresidues Ser(116) and Thr(497) [163]. Epinephrine also activates the small G protein Rac1 and protein kinase A which can be antagonized by the β3-adrenoceptor antagonist, SR59230A. Moreover, regular exercise training can cause eNOS to be less coupled and elevate NOS-dependent superoxide levels in β3-adrenoceptor knock-out mice [164]. It has been shown that supplementing those β3-adrenoceptor knockout mice with the eNOS coupler BH4 during the exercise training protocol can prevent an attack of MI. Thus, regular exercise has a beneficial role by protecting the heart in the setting of myocardial ischemia/reperfusion injury via activation of the eNOS protein through a β3-adrenoceptor-AMP-activated protein kinase signaling pathway [164].

### eNOS and the Parasympathetic Autonomic Nervous System

The PANS role on the CVS largely includes the functions of the vagus nerve, the neurotransmitter acetylcholine (ACh), and muscarinic receptors, particularly the M2-subtype within the SA node in the heart. Both the SANS and PANS have their mutual influence on the heart; activation of the SANS increases HR while stimulation of the PANS causes bradycardia. The PANS also has parasympathetic fibers that innervate small blood vessels outside of the heart, however, the primary function of the PANS is limited to controlling the cardiac function via regulating the HR and maintaining a vascular tone [153]. Although stimulation of the PANS causes bradycardia, it plays a minor role in regulation of blood pressure when compared with the SANS, and thus there are few studies that target the PANS as a treatment strategy for hypertension and other CVD. Nevertheless, a decrease in PANS activity is associated with essential hypertension in humans. A dysfunctional PANS can reduce HR variability and is associated with CVD such as MI, heart failure, and cerebrovascular accidents [165]. In addition, exercise-induced potentiation of basal parasympathetic tone may have a beneficial effect in alleviating hypertension-associated end-organ damage [165].

In order to stimulate the PANS, there have been some experiments where electrical nerve stimulators are implanted on the cervical vagus nerve to chronically stimulate it. Moreover, there is a hypothesis that this chronic vagal nerve stimulation (VNS) can reduce the progression of end-organ damage in hypertensive rats. Chronic VNS only affects endothelial function and aortic stiffening but has no role in cardiac functions or vascular hypertrophic changes166. The study also demonstrates that the gradual reduction in NO-dependent relaxation of the long posterior ciliary artery in sham-stimulated rats that are on a high-salt diet is prevented in rats with chronic VNS, and this improved endothelial function is attributed with a greater activity and phosphorylation of the eNOS protein [166]. The PANS neurotransmitter ACh produces an indomethacin-sensitive relaxation in the mesenteric arteries of knockout NOS (−/−) mice while the resting membrane potential of vascular smooth muscle cells derived from the coronary arteries is considerably less negative in the eNOS (−/−) mice [167]. The activity/bioavailability of coupled eNOS and subsequent generation of NO can be either initiated or enhanced by several stimuli that include Ach [168]. In hypertensive patients or even people with prehypertension, the NO-mediated relaxation in response to ACh is blunted [169]. This is also true for normotensive and hypertensive rats suggesting that the reduced NO bioavailability can be attributed to a dysfunctional eNOS-derived decreased NO production and/or an increased NO degradation [170]. It is known that regular exercise is a nonpharmacological therapy for not only prevention but also for treatment of CVD because of its beneficial milieu in improving vascular function. Indeed, exercise training can induce eNOS coupling that in turn, can restore relaxation in coronary arteries of heart failure rats [170]. Overall, the interaction between the PANS and its neurotransmitter ACh and the eNOS isoform contributes to healthy cardiovascular functions.

## Endothelial Nitric Oxide Synthase and Cardiovascular Autonomic Reflexes

The cardiovascular autonomic reflexes are responses that have profound physiological impact on the cardiovascular structures and functions either through direct neural innervation or via modulating the release of chemicals/hormones or substances such as epinephrine, renin, and anti-diuretic hormone. Cardiovascular reflexes regulate and maintain bodily homeostasis during normal day-to-day activities like sleep, exercise, walking, running, rage, anger, or other situations. The two main cardiovascular autonomic reflexes are the baroreflex and the Exercise Pressor Reflex.

### eNOS and Baroreflex

The arterial baroreceptor reflex, or baroreflex, is the most important mechanism for minute-to-minute regulation and maintenance of blood pressure that buffer the continuous fluctuations/changeability of blood pressure due to posture, exercise, pain, emotion, and other circumstances. Several neurological situations can modulate the central and/or peripheral baroreflex mechanisms that in turn, may manifest with paroxysmal hypertension, a reflex syncope, or even neurological orthostatic hypotension. One example of the baroreflex-mediated cardiovascular homeostasis is that if the blood pressure rises, the PANS activity is increased along with a decrease in the SANS reactivity. The major baroreceptor reflex pathway exists within the medullary region of the brainstem, i.e., the NTS, the RVLM, and the CVLM [157]. The overexpression/activity of eNOS within the RVLM or NTS decreases blood pressure, HR, and renal sympathetic nerve activity that are augmented in stroke-prone spontaneously hypertensive rats [150,158]. An increase in eNOS-derived NO production within the RVLM significantly improves the impaired baroreflex regulation of HR in stroke-prone spontaneously hypertensive rats and that one of the mechanisms is related to cardiac sympatho-inhibitory effects of NO [150]. It is now well-established that the RVLM neurons can regulate the activity of the SANS via both baroreflex-dependent and baroreflex-independent mechanisms. These corraborate with other data on central and peripheral neural mechanisms that regulate arterial baroreceptor reflex and mechanisms of adaptation and resetting of the baroreceptor reflex [166,171]. However, endogenous NO production by the eNOS protein within the RVLM is impaired or insufficient that can compensate for the abnormal baroreflex function in hypertension, although it is known that the RVLM neurons receive a tonic GABAergic input that is partially dependent on baroreceptor inputs and partially independent [171].

The baroreceptor-regulated blood pressure buffering effect of endogenous NO is shown to be mediated by the eNOS protein and that eNOS-derived endothelial NO is most effective in buffering blood pressure oscillations via modulating the baroreflex mechanisms in experiments with conscious wild-type and eNOS mutant mice [172]. However, the same study cannot find any differences in baroreceptor reflex sensitivity between eNOS mutant and wild-type control mice; thus, the question is why the baroreceptor reflex does not cancel the enhanced blood pressure changeability in eNOS knockout mice. It may be possible that the higher variability in blood pressure changes as observed in eNOS knockout mice is partially compensated for by the baroreceptor reflex and is dependent on a functional eNOS system. An endogenous eNOS expression or activity within the NTS has been suggested to play a role in long-term sensitivity of baroreceptor reflex gain and a study has determined that chronic inhibition of eNOS activity within the NTS enhances baroreceptor reflex in experiments with conscious rats [173]. Another report using adenoviral-mediated gene transfer of a dominant negative protein to genetically disable eNOS in the NTS shows that the effect of an administration of AngII on the baroreceptor reflex is blocked, which further validates that the action of AngII is mediated by activation of the eNOS protein [174]. Indeed, the NTS modulates both the baroreceptor and the Exercise Pressor Reflex via a NO-dependent mechanism within the NTS [175]. Furthermore, local application of the non-specific NOS inhibitor, NG-nitro-L-arginine methyl ester (L-NAME), to the carotid sinus abolishes the inhibition of baroreceptor activity and the increases in carotid artery diameter in eNOS-transduced carotid sinuses suggesting that adenoviral-mediated gene transfer of eNOS to carotid sinus adventitia causes a NO-dependent inhibition of the baroreceptor mechanism, including a reset of the baroreceptor reflex in response to high blood pressure [176]. Because the baroreflex plays a critical role in regulating/controlling short term blood pressure changes, there are polymorphisms identified within the eNOS gene, i.e., the CYP11B2 and the B2R genes, that are associated with 16% of the variation in baroreflex sensitivity [177].

### eNOS and the Exercise Pressor Reflex

**Figure 4:**
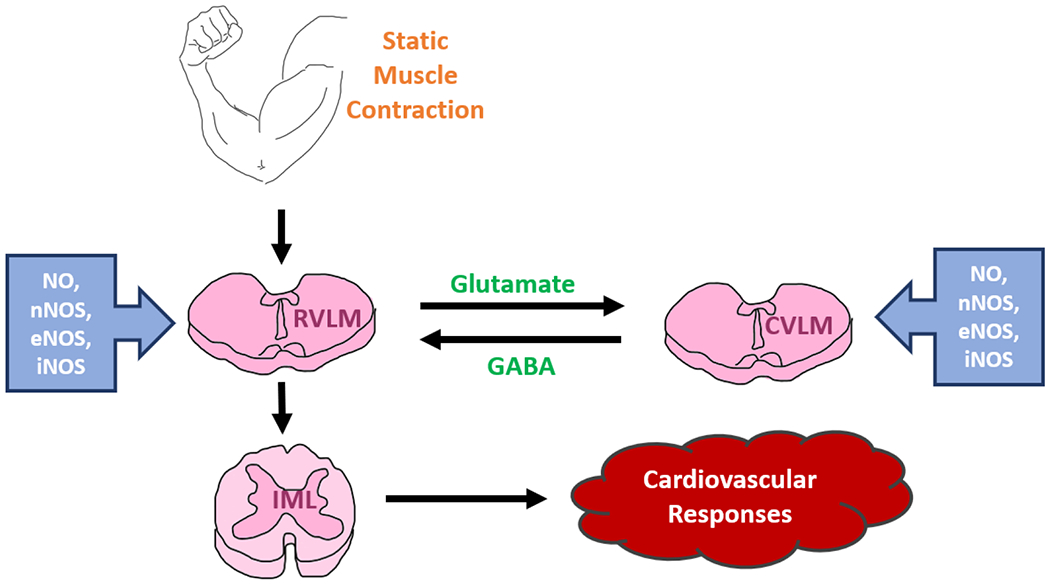
A summary of the Exercise Pressor Reflex arc reflecting major neurotransmitters and regions of the brain that are involved. Abbreviations: NO - nitric oxide; eNOS – endothelial nitric oxide synthase; iNOS – inducible nitric oxide synthase; nNOS – neuronal nitric oxide synthase; RVLM – rostral ventrolateral medulla; CVLM – caudal ventrolateral medulla; IML – intermediolateral column of the spinal cord.

Static exercise or static muscle contraction raises MAP, HR, myocardial contractility, cardiac output, and sympathetic nerve activity [139,157,178,179]. The exercising muscles activate the group III and IV muscle afferents within the spinal cord, transmit the information to different parts of the brain, particularly to the neurons in the medulla oblongata of the brain stem, however, the main areas of integration of the signals are the RVLM or the pressor region and the CVLM or the depressor area. A plethora of molecular mechanisms, neurotransmitters, receptors, and reciprocal pathways are involved within these two RVLM and CVLM areas as well as other higher regions of the brain that are collectively known as the “central command”. Pharmacological manipulations by L-arginine, the NO precursor, or by nNOS, iNOS, and eNOS antagonists are involved in the modulation of the Exercise Pressor Reflex [1,180]. A brief schematic diagram of the Exercise Pressor Reflex is shown in (Figure 4).

The nNOS isoform within the RVLM and the CVLM differentially modulates cardiovascular responses during static exercise [139,152]. Moreover, the modulation of the exercise pressor reflex by nNOS is attributed to differential alterations in glutamate and GABA neurotransmission within the RVLM and CVLM, and that blockade of nNOS can also modify the expression of the nNOS isoform within the RVLM and CVLM [181]. A similar modulation of the exercise pressor reflex by the iNOS isoform via glutamate, GABA, and expression of the iNOS protein is regulated within the RVLM and CVLM1. The eNOS protein has also been implicated in the mechanism of the exercise pressor reflex. Overexpression of eNOS protein by elevated eNOS causes hypotension and bradycardia suggesting its role in decreasing the activity of the SANS150. Moreover, overexpression of eNOS within the RVLM attenuates MAP, HR, and sympathetic nerve activity, and these effects are more pronounced in stroke-prone hypertensive rats. An increase in blood eNOS levels decreases plasma cholesterol values and offers protection against atherosclerosis or other CVD, conditions that can be prevented by regular exercise [182]. The eNOS differentially alters MAP and HR changes in response to static exercise via modifications of glutamate and GABA concentrations within the RVLM and CVLM, and that eNOS antagonism by pharmacological agents also modulates the expression of the eNOS isoform within the RVLM and CVLM [1,131]. Administration of an eNOS blocker, L-NIO at a dose of 10 μM, into the RVLM in anesthetized rats potentiates the increases in MAP and HR responses during static muscle contraction and opposite effects happen when the drug is microdialyzed into the CVLM152. The 10 μM L-NIO did not change the basal blood pressure or HR, however, a higher 50 μM dose of L-NIO administered into the RVLM increases resting basal blood pressure. Simultaneously, microdialysis of L-NIO into bilateral RVLM for 120 minutes attenuates the abundance/expression of eNOS as measured from the same area183. Thus, the autonomic and cardiovascular reflex mechanisms within the medulla oblongata to achieve a physiological homeostasis are regulated via a balance of an excitatory and an inhibitory interaction among the eNOS, nNOS, and iNOS proteins and their synthesis of NO, in addition to glutamatergic and GABAergic neurotransmission [1,139]. However, other molecular and cellular changes may also occur within the VLM following focal cerebral ischemic injury that can further elucidate the pathogenesis, circulatory manifestations, and treatment strategies of stroke, including the potential benefits of exercise. Exercise is recommended after stroke as a part of rehabilitation, yet the interaction between exercise and brainstem mechanisms mediating cardiovascular activity is still poorly understood. It should be noted here that the NTS is also involved in the modulation of the Exercise Pressor Reflex functions via NO/cGMP pathways within the NTS [175].

## Endothelial nitric oxide synthase and Cardiovascular Diseases

Numerous studies and research are being performed to delineate the mechanisms of the nNOS/eNOS/iNOS/NO/cGMP/cGC pathways and how this highly reactive gas known as NO that can change into a free radical is involved in regulation and pathogenesis of the CVS (Figure 2) [184].

### eNOS and Hypertension

Hypertension is typically asymptomatic. However, some people with hypertension may have headaches, lightheadedness, vertigo, tinnitus, altered vision or fainting episodes. Hypertension with certain specific additional signs and symptoms may suggest secondary hypertension, i.e., hypertension due to an identifiable cause such as pheochromocytoma or kidney disease. Hypertension occurs in ~10% of pregnancies and can be associated with pre-eclampsia which may be either asymptomatic or with manifestations of headache, visual disturbance (“flashing lights”), vomiting, pain over the stomach, and swelling. The eNOS plays a crucial role in blood pressure regulation and that it is mediated via eNOS-derived NO production resulting in vasodilation through cGMP-dependent protein kinase (PKG) activation within the vascular smooth muscle cells (VSMCs; Figure 3) [66,67,185]. Essential hypertensive patients do experience dysfunction of NO-mediated vasodilatation in several arterial trees such as the brachial, coronary, and renal arteries, and that the transport of L-arginine is dysfunctional in hypertensive or even normotensive human subjects who have a family history of essential hypertension [186]. The importance of L-arginine cannot be underestimated because oral supplementation of L-arginine benefits in restoring endothelial function in hypertensive patients by modulating the release of neuroendocrine hormones such as catecholamines, altering plasma renin-AngII activity, and decreasing plasma aldosterone levels [187]. Measurement of MAP in the eNOS deficient mice shows a 20 mmHg higher MAP when compared to its wild type variants [145]. Thus, a thorough understanding of the molecular mechanisms of eNOS dysfunction in hypertension may lead to further research and development of novel therapeutic agents that will manage hypertension, the “Silent Killer” disease. To elaborate further, there are several mechanisms that have been proposed for eNOS-derived NO deficiency in hypertension. The eNOS is important for a healthy vasculature that not only prevents hypertension but also stops the process of atherosclerosis [188]. A deficiency of NO by superoxide anion will cause endothelial dysfunction and hypertension. All of the following e.g., NADPH oxidase (NOX1), xanthine oxidase (XO), uncoupled eNOS, mitochondria, and cyclooxygenase (COX) produce ROS [189]. COX-2 generates oxidative stress within resistance arteries of patients suffering from essential hypertension that in turn, may lower the bioavailability of NO [190]. Furthermore, COX-2-derived prostaglandin F(2α) is linked to endothelial dysfunction in renovascular hypertensive rats. Finally, the ROS produced by NOX is shown to increase expression of COX-2 via a p38 MAPK-dependent mechanism that in turn, causes uncoupling of the eNOS protein [191].

Angiotensin II causes vasoconstriction and an increase in blood pressure, and its plasma concentrations are consistently elevated in hypertensive patients [192] and AT1R antagonists such as Valsartan are highly effective in lowering blood pressure to normal levels. One study demonstrates that eNOS uncoupling leads to the development of hypertension in mice receiving an infusion of AngII [193]. Aneurysm mediated by eNOS uncoupling can be reversed by the administration of folic acid in AngII-infused apolipoprotein E (apoE) null mice because of the restoration of endothelial dihydrofolate reductase (DHFR) expression and activity [194]. Because the uncoupling of eNOS is a pathogenesis of essential hypertension, any therapeutic target that is able to restore eNOS coupling activity in blood vessels can be a part of the treatment protocol for hypertensive patients. Anticholesterolemic medications such as statins, anti-hypertensive drugs such as losartan, and even vitamin D3 are beneficial to a healthy vasculature. BH4 deficiency plays a key role in determining eNOS uncoupling-dependent hypertension and supplementation with BH4 may have a beneficial therapeutic effect that will restore endothelial dysfunction in hypertensive patients via endothelium-dependent vasodilation [195]. Moreover, ascorbic acid or vitamin C can maintain BH4 levels during situations of increased vascular oxidative stress, and that this vitamin C can prevent uncoupling of eNOS by ONOO-196. Further, in genetic models of hypertensive rat, vitamin C can improve endothelial function via restoration of the eNOS isoform [197]. Finally, oral administration of BH4 can attenuate the production of vascular ROS and lowers blood pressure in DOCA-salt hypertensive rats [198].

### eNOS, Arteriosclerosis, and Myocardial Infarction

Myocardial infarction or MI is commonly known as heart attack and happens when there is a reduced coronary blood flow (ischemia) or a complete block in the blood supply to a part of the heart resulting in damage or necrosis of the heart muscle or myocardium [199]. The most common symptom is chest pain or discomfort which may radiate to the shoulder and arm that may end up with heart failure, an irregular heartbeat such as atrial fibrillation, cardiogenic shock or cardiac arrest. Most MIs occur due to coronary artery disease and a complete blockage of any branch of the coronary artery due to a rupture of an atherosclerotic plaque is commonly the underlying mechanism of an MI. Thus, MI and atherosclerosis are very closely related to each other [199].

The role of oxidative stress in the progression and pathogenesis of CVD has been shown in an experimental animal model of hypercholesterolemia [200]. Other studies using genetic animal models such as knockout mice also report how ROS is critically engaged in the development, pathogenesis, and consequences of several CVD like arteriosclerosis that may lead to an event of MI [201]. In mice, genetic deletion of the NADPH oxidase subunit p47phox can restore NO bioavailability in coronary arteries, prevents the production of ROS, and at the same time benefits cardiovascular functions by increasing the survival rate by 20% in the event of post-MI circumstances [202]. Deletion of the NADPH oxidase subunits p47phox reverses hypertension to normal values and restores endothelial health in AngII-infused hypertensive mice [203]. In addition, in mice with a partial deletion of the mitochondrial superoxide dismutase (MnSOD+/−) there is an elevated susceptibility of mitochondrial oxidative stress along with a dysfunctional endothelial cell layer in the coronary arteries [204]. Likewise, a totally deficient mitochondrial superoxide dismutase enzyme will make mice at a higher risk of endothelial dysfunction, and deletion of the glutathione peroxidase-1 will cause significant atherosclerosis and plaque formation leading to thrombosis in ApoE−/− mice [205]. If NOX1 has an increase in its activity and is overexpressed, there will be elevated blood pressure [206]. Several interleukins are also involved in the link between eNOS and arteriosclerosis. For example, IL-17A plays an active role in vascular inflammation that will lead to atherosclerosis and hypertension, including MI [207]. Another study also shows that IL-17A plays a critical role in the onset and progression of AngII infusion-induced vascular dysfunction [208]. Together, many studies demonstrate that eNOS has a crucial role in causing oxidative stress that in turn, may lead to the development of several CVD such as arteriosclerosis and MI.

Myocardial infarction causes significant LV remodeling, cardiomyocyte swelling or hypertrophy, a decrease in capillary tree, an increase in fibrosis, apoptosis, necrosis, LV systolic dysfunction, and LV diastolic imbalance. The beneficial effects of regular exercise for a healthy CVS are well established and even exercise or static exercise is a part of the treatment protocol following a cardiovascular injury including post-MI. There are numerous studies of the beneficial effects of exercise including in a post-MI situation, and there is evidence that this positive effect of exercise is mediated via an increase in eNOS activity. Exercise training by eNOS +/+ MI mice totally reverses the fibrosis and apoptosis in the myocardium and decreases LV systolic dysfunction that are not seen in eNOS +/− and eNOS −/− mice [113]. AMP-activated protein kinase (AMPK) is a protein kinase-sensing metabolite and if activated after MI it becomes an endogenous protective signaling mechanism; and pre-administration of metformin (an oral diabetes type 2 medicine) has a cardio-protective role against MI that is mediated via the AMPK-eNOS-mediated signaling cascade [209]. Myocardial infarction causes death of cardiomyocytes due to a lack of blood supply and may end up as a fibrotic scar in the myocardium. Scientists have developed an injectable conductive hydrogel encapsulating plasmid DNA-eNOS adipose derived stem cells (ADSCs) for the treatment of MI and the results are significant increase of ejection fraction (EF), shortened QRS interval in the EKG, smaller infarction size, lessened fibrosis area, a higher density of capillary vasculature, and a significant improvement of cardiovascular hemodynamics [210]. The heat shock protein, HSPA12B, has been shown to attenuate cardiac dysfunction and reduces cardiac remodeling following MI via an eNOS-dependent mechanism [120]. In addition, inhibition of TGF-β1 (transforming growth factor) signaling due to eNOS gene transfer can facilitate ventricular remodeling following MI that is mediated by angiogenesis and a stoppage of apoptosis [211]. The chemical compound resveratrol (RSV, 3, 5, 4-trihydroxystilbene), a naturally polyphenolic phytoalexin, has been shown to attenuate myocardial ischemia/reperfusion injury mediated via up-regulation of vascular endothelial growth factor B (VEGF-B)/AMPK/eNOS/NO signaling mechanisms in isolated rat heart preparations [212]. Finally, β3-adrenergic receptors have been identified as modulators of heart function and there have been proposals that these receptors can be potential therapeutic targets for the management of CVD. Indeed, β3-adrenoreceptor stimulation has been shown to protect against MI-induced tissue death via activation of the eNOS protein [213]. Overall, there have been numerous studies that depict the use of different pharmacological manipulations in order to delineate the role of the eNOS protein following an episode of MI. The potential for an eNOS-related pharmacological therapy protocol appears to be a promising subject in the future.

### eNOS and Stroke

Cerebrovascular stroke leads to numerous molecular, neurochemical, and cellular changes such as massive release of glutamate and activation of inflammatory cytokines, delayed neuronal cell death and apoptosis, inflammation and peri-infarct depolarizations within the peri-infarct zone or ischemic penumbra, however, neuronal death following ischemia is due to the massive generation of free radicals like NO [1,139]. The production of NO from the neural tissues within the brain following an event of stroke is dependent on differential activation of all 3 isoforms of the NOSs enzymes: nNOS, iNOS and eNOS, however, exactly how many molecules of NO are generated from one molecule of each isoform are still unknown. In addition, it is yet to be determined exactly how much is the expression of each NOS protein in different parts of the affected brain following a stroke which in turn, may determine how many neurons will be either protected or destroyed. Because the focus of this review is only on the eNOS isoform, the following paragraphs will discuss the past and recent literature regarding the role of eNOS in the pathogenesis of stroke.

While the transdermal glyceryl trinitrate is used in acute stroke, there is no evidence to recommend the use of the eNOS protein or drugs that only modifies eNOS in stroke patients. Thus, most of the investigations for the role of eNOS in stroke are performed using experimental animals. Several animal studies show that during the early stages of an ischemic brain injury, a surge in eNOS-derived NO release protects the neurons from death or apoptosis mediated via vasodilatation and inhibition of microvascular aggregation [214]. Thus, the role of eNOS has been extensively studied using animal models of cerebral ischemia, and it has been shown that a transient 90-minute left-sided middle cerebral artery occlusion (MCAO)-induced focal ischemia followed by 24 hours of reperfusion significantly attenuates eNOS expression within the left or ipsilateral RVLM when compared to the abundance of eNOS within the contralateral RVLM [183]. At the same time, expression of the eNOS protein derived from the ipsilateral CVLM following a left sided MCAO is significantly augmented when compared to the contralateral CVLM [183]. It can be suggested that temporary ischemic stroke can alter ipsilateral eNOS protein abundance differentially within the VLM, which may result in a decreased rise in blood pressure in response to static muscle contraction. Similar results were observed when measuring the abundance of the nNOS protein, while there were opposite expressions of the iNOS enzyme within the RVLM and CVLM in stroked rats [139]. Therefore, eNOS plays a significant role in modulating cardiovascular responses and in the pathogenesis of stroke within the brainstem. A search on the literature shows numerous other investigations regarding the role of the eNOS enzyme following ischemic stroke in animal models and are discussed in subsequent paragraphs.

Overall, NO within the brainstem plays a significant physiological role in modulating cardiovascular responses during static muscle contraction. Inhibition of nNOS, eNOS, or iNOS within the RVLM differentially alters glutamate and GABA neurotransmission that in turn, results in modulation of cardiovascular responses during static exercise. In contrast, blockade of different NOS isoform activities within the CVLM causes dif-ferent alterations in cardiovascular responses to muscle contraction, mediated via changes in glutamate and GABA levels within the CVLM. Thus, the NOS enzyme within the medulla appears to provide an interactive mechanistic role in glutamatergic and GABAergic neurotransmission during integration of cardiovascular functions during static exercise. Further, blockade of the nNOS isoform within the medulla modifies expression of the nNOS protein and modulates cardiovascular responses during static exercise. However, the molecular roles of these three isoforms on NO production and/or protein expression/activity remain to be investigated. Nevertheless, NO-mediated mechanisms are involved in cardiovascular homeostasis and its adaptation to exercise.

The eNOS has been a key item in molecular stroke research for the last two decades because not only it can ameliorate acute cerebral ischemic injury but also can promote early recovery following cerebral ischemia via regulating cerebral blood flow, promoting homeostasis, reducing inflammation, and generating angiogenesis as well as neurogenesis. Endothelial NOS expression increases very early after a cerebral ischemic insult. In animal models of stroke, intravenous administration of the eNOS substrate, L-arginine, during ischemia has been shown to be neuroprotective [215]. Moreover, several pharmacological protocols using medications such as HMG-CoA-reductase inhibitors or commonly referred to as statins, AT2R antagonists, and Ca^2+^-channel blockers increase the expression/activity of eNOS, that in turn, can benefit CVD via attenuating endothelial dysfunction and promoting global and regional blood flow [216]. There is an enhanced incidence of stroke in patients who are homozygous for the eNOS polymorphism on exon 7 (894G > T) [217]. Thus, further investigations using the genetic approach as a translational model are crucial to fully characterize the role of eNOS in stroke of even a transient attack of cerebral ischemia in humans. The cerebral vascular tree is geared to rapidly respond to any alterations in systemic blood pressure in order to maintain cerebral blood flow homeostasis. The cerebral arteries and arterioles vasoconstrict when there are elevations of blood pressure and dilate in response to hypotension. As mentioned previously, endothelial cells-derived NO is known to regulate the basal vascular tone. Stroke or cerebral ischemia happens when there is a loss of blood flow and hence a major goal in the management of ischemic stroke is to rapidly restore the blood circulation that can reduce further neuronal death. The infusion of L-arginine or other NO donors following an acute event of ischemia reduces infarct size by improving blood circulation within the penumbra region surrounding the infarct [215]. In addition to NO donors, AT2R blockers and statins or regular physical exercise also provide neuroprotective effects via eNOS/NO/cGMP-dependent path-ways as shown by studies using acute and chronic experimental stroke animal models [218]. Regular exercise and increase in physical activity also improve endothelial function which enhances vasodilation and vasomotor function [219]. Identifying the mechanisms underlying the cardiovascular benefits of exercise in stroke patients is crucial for improving treatment strategies for stroke. It is important to understand the mechanisms within the brainstem that integrate the function of the nervous and CVS during static exercise and following cerebral ischemia. Also, it is critical to uncover the mechanisms by which exercise training in stroke patients can improve the compromised relationship between the nervous system and the CVS.

Stroke-prone spontaneously hypertensive rats exhibit an attenuated expression of the eNOS protein within the cerebral cortex and within the endothelium [220]. Furthermore, AT2R blockade potentiates cerebral circulation and can lessen further neuronal death [221]. Statin medications have fibrinolytic and antithrombotic effects and attenuate aggregation of platelets mediated via an elevated eNOS-expression-derived NO formation in MCAO rats222. Another mechanism by which an increased expression/activity of eNOS is beneficial in stroke patients is by promoting an increased cerebral circulation through the process of angiogenesis from precursor stem cells that are increased in ischemic brain injury, and that NO works as a messenger/neuromodulator that promotes endothelial cell proliferation and migration. This angiogenesis is also regulated by several growth factors such as TGF-β2, Platelet-derived growth factor (PDGF), Vascular endothelial growth factor (VEGF), and Basic fibroblast growth factor 2 (BFGF-2), which are highly expressed following a cerebral ischemic insult and that eNOS can stimulate angiogenesis by releasing low concentrations of NO [223]. Overall, eNOS preserves collateral blood flow following an ischemic brain injury and thereby prevents further neuronal damage making it a key protein for future use in stroke research because eNOS has the ability to not only ameliorate acute ischemic insult but also promote early recovery. The efficacy of NO is studied in humans with the idea of managing hypertension in acute stroke and the result is that transdermal glyceryl trinitrate lowers blood pressure but does not improve the functional outcome (The RNOS Trial Investigators, 2016). Finally, the drug Rapamycin®, (also known by its generic name, Sirolimus) is a natural product isolated from Streptomyces hygroscopicus that is found on the island of Rapa Nui inhabitants (Polynesian inhabitants) of Easter Island in the Pacific Ocean near Chile [224]. This Rapamycin® is a clinically approved mammalian target of rapamycin inhibitor that has been shown to possess neuroprotection in animal models of ischemic stroke. However, the mechanism of Rapamycin®-induced neuroprotection is still unknown although sirolimus-eluting stents are safe and effective in preventing restenosis in de novo coronary artery lesions. A recent study has shown that Rapamycin® significantly increases collateral perfusion and reperfusion of the cerebral blood vessels in both Wistar and comorbid spontaneously hypertensive rats that appear to be mediated via augmenting the activation of eNOS [225]. This suggests that Rapamycin® can be used in the treatment protocol in patients with stroke as this drug increases collateral blood flow and also in patients who have undergone thrombolysis or thrombectomy by improving cerebral blood flow circulation [225]. Manipulations of the eNOS protein with its agonists/antagonists administered either locally or systemically and their effects on cardiovascular function following stroke should be further explored in order to ascertain possible therapeutic benefits/disadvantages that in turn, may lead to the development of drugs for the prevention or treatment of stroke. In summary, eNOS has an important in the pathogenesis, prevention, and management in a situation of ischemic brain injury or stroke.

## Highlights and Conclusion

The intricate and critical relationships between the eNOS protein and the CVS in both physiological and in the pathogenesis of a diverse array of CVD have been discussed. The eNOS isoform induces autocrine and paracrine signaling factors that modulate blood flow, circulation, and neural activity via the production of NO. The resulting vasodilation elicited from the catalytic creation of NO allows the maintenance of tonicity and blood flow within the circulation. The detrimental impacts of eNOS uncoupling are involved in the pathogenesis of numerous CVD.

In conclusion, most of the plethora of the roles of eNOS in both physiological and pathological states of the CVS that are related to the eNOS gene and chromosome 7, eNOS uncoupling, differential regulations via receptors- and drug-mediated pathways, cardiovascular autonomic reflexes, baroreflex, and the Exercise Pressor Reflex have been elaborated. Moreover, the interrelated relationship between eNOS, the CVS, the CNS, and the pathogenesis of several CVD such as hypertension, atherosclerosis, MI, and stroke that may happen because of disrupted or reduced eNOS activity are critically evaluated. Further research on the development of drugs that can modulate or affect eNOS expression/activity, eNOS uncoupling, and related regulatory mechanisms can contribute to the better understanding of the relationship between eNOS protein and the CVS/CVD. Finally, the pathogenesis of several CVD related to eNOS should be extensively clarified not only to prevent diseases like hypertension and stroke but also be used as a foundation of their treatment and follow-up.

## References

[1] Ally A, Powell I, Ally MM, Chaitoff K, Nauli SM (2020) Role of neuronal nitric oxide synthase on cardiovascular functions in physiological and pathophysiological states. Nitric Oxide 102: 52–73. 32590118 10.1016/j.niox.2020.06.004 PMC7375925

[2] Li Q, Youn JY, Cai H (2015) Mechanisms and consequences of endothelial nitric oxide synthase dysfunction in hypertension. J Hypertens 33(6): 1128–1136. 25882860 10.1097/HJH.0000000000000587 PMC4816601

[3] Zhang T, Guo D, Zheng W, Dai Q (2021) Effects of S1PR2 antagonist on blood pressure and angiogenesis imbalance in preeclampsia rats. Mol Med Rep 23(6): 456. 33880585 10.3892/mmr.2021.12095

[4] Eid SA, Hinder Lucy M, Zhang Hongyu, Eksi Ridvan, Nair Viji, (2021) Gene expression profiles of diabetic kidney disease and neuropathy in eNOS knockout mice: Predictors of pathology and RAS blockade effects. FASEB J 35(5): e21467. 33788970 10.1096/fj.202002387R PMC10202027

[5] Ninchoji T, Love Dominic T, Smith Ross O, Hedlund Marie, Vestweber Dietmar, (2021) eNOS-induced vascular barrier disruption in retinopathy by c-Src activation and tyrosine phosphorylation of VE-cadherin. Elife 10: e64944. 33908348 10.7554/eLife.64944 PMC8087444

[6] Garcia Martin E, Muñoz Santiago Navarro, Rodriguez Christopher, Serrador Mercedes, Navarro Hortensia Alonso, (2020) Association between endothelial nitric oxide synthase (NOS3) rs2070744 and the risk for migraine. Pharmacogenomics J 20(3): 426–432. 31792366 10.1038/s41397-019-0133-x

[7] Guo C, Jiang Jun, Cheng Bo, Xie Libo, Lin Haocheng, (2020) Low androgen status inhibits erectile function by up-regulating the expression of P2X receptors in rat corpus cavernosum. Andrologia 52(7): e13627. 32352595 10.1111/and.13627

[8] Porembskaya O, Toropova Yana, Tomson Vladimir, Lobastov Kirill, Laberko Leonid, (2020) Pulmonary Artery Thrombosis: A Diagnosis That Strives for Its Independence. Int J Mol Sci 21(14):5086. 10.3390/ijms21145086 PMC7404175 32708482

[9] Wang Y, Branicky R, Noe A, Hekimi S (2018) Superoxide dismutases: Dual roles in controlling ROS damage and regulating ROS signaling. J Cell Biol 217(6): 1915–1928. 29669742 10.1083/jcb.201708007 PMC5987716

[10] Flohe L (2020) Looking Back at the Early Stages of Redox Biology. Antioxidants (Basel) 9(12): 1254. 10.3390/antiox9121254 PMC7763103 33317108

[11] Fridovich I (1995) Superoxide radical and superoxide dismutases. Annu Rev Biochem 64: 97–112. 7574505 10.1146/annurev.bi.64.070195.000525

[12] Fridovich I (1978) Superoxide dismutases: defence against endogenous superoxide radical. Ciba Found Symp 6-8(65): 77–93. 10.1002/9780470715413.ch6 225147

[13] Douki T, Cadet J (1996) Peroxynitrite mediated oxidation of purine bases of nucleosides and isolated DNA. Free Radic Res 24(5): 369–380. 8733941 10.3109/10715769609088035

[14] Czapski G, Goldstein S (1995) The role of the reactions of .NO with superoxide and oxygen in biological systems: a kinetic approach. Free Radio Biol Med 19(6): 785–794. 10.1016/0891-5849(95)00081-8 8582651

[15] Rai H, Parveen F, Kumar S, Kapoor A, Sinha N (2014) Association of endothelial nitric oxide synthase gene polymorphisms with coronary artery disease: an updated meta-analysis and systematic review. PLoS One 9(11): e113363. 25409023 10.1371/journal.pone.0113363 PMC4237457

[16] Seremak-Mrozikiewicz A, (2011) The significance of −786T > C polymorphism of endothelial NO synthase (eNOS) gene in severe preeclampsia. J Matern Fetal Neonatal Med 24: 432–436. 20822330 10.3109/14767058.2010.511329

[17] Niu W, Qi Y (2011) An updated meta-analysis of endothelial nitric oxide synthase gene: three well-characterized polymorphisms with hypertension. PLoS One 6(9): e24266. 21912683 10.1371/journal.pone.0024266 PMC3166328

[18] Dai B, Liu T, Zhang B, Zhang X, Wang Z (2013) The polymorphism for endothelial nitric oxide synthase gene, the level of nitric oxide and the risk for pre-eclampsia: a meta-analysis. Gene 519(1): 187–193. 23375994 10.1016/j.gene.2013.01.004

[19] Shoukry A, Shalaby Sally M, Abdelazim Shymaa, Abdelazim Marwa, Ramadan Ayman, (2012) Endothelial nitric oxide synthase gene polymorphisms and the risk of diabetic nephropathy in type 2 diabetes mellitus. Genet Test Mol Biomarkers 16(6): 574–579. 22313046 10.1089/gtmb.2011.0218

[20] Eroz R, Bahadir A, Dikici S, Tasdemir S (2014) Association of endothelial nitric oxide synthase gene polymorphisms (894G/T, −786T/C, G10T) and clinical findings in patients with migraine. Neuromolecular Med 16: 587–593. 24845269 10.1007/s12017-014-8311-0

[21] Nakayama M, Yasue H, Yoshimura M, Shimasaki Y, Kugiyama K, (1999) T-786-->C mutation in the 5’-flanking region of the endothelial nitric oxide synthase gene is associated with coronary spasm. Circulation 99(22): 2864–2870. 10359729 10.1161/01.cir.99.22.2864

[22] Hendrix P, Hendrix P, Foreman PM, Harrigan MR, Fisher WS, (2017) The role of endothelial nitric oxide synthase −786 T/C polymorphism in cardiac instability following aneurysmal subarachnoid hemorrhage. Nitric Oxide 71: 52–56. 29079038 10.1016/j.niox.2017.10.008

[23] Hendrix P, Foreman Paul M, Harrigan Mark R, Fisher Winfield S 3rd, Vyas Nilesh A, (2017) Endothelial Nitric Oxide Synthase Polymorphism Is Associated with Delayed Cerebral Ischemia Following Aneurysmal Subarachnoid Hemorrhage. World Neurosurg 101: 514–519. 28254540 10.1016/j.wneu.2017.02.062

[24] Ko NU., Rajendran Pam, Kim Helen, Rutkowski Martin, Pawlikowska Ludmila, (2008) Endothelial nitric oxide synthase polymorphism (−786T->C) and increased risk of angiographic vasospasm after aneurysmal subarachnoid hemorrhage. Stroke 39(4): 1103–1108. 18309169 10.1161/STROKEAHA.107.496596 PMC2650384

[25] Abbasi H, Dastgheib Seyed Alireza, Hadadan Amaneh, Zarchi Mojgan Karimi, Javaheri Atiyeh, (2020) Association of Endothelial Nitric Oxide Synthase 894G > T Polymorphism with Preeclampsia Risk: A Systematic Review and Meta-Analysis based on 35 Studies. Fetal Pediatr Pathol 40(5): 1–16. 10.1080/15513815.2019.1710880 31920131

[26] Kumar GR, Spurthi Kondapalli Mrudula, Kumar Gundapaneni Kishore, Aiyengar Tupurani Mohini, Chiranjeevi Padala, (2016) Genetic polymorphisms of eNOS (−786T/C, Intron 4b/4a & 894G/T) and its association with asymptomatic first degree relatives of coronary heart disease patients. Nitric Oxide 60: 40–49. 27613099 10.1016/j.niox.2016.09.001

[27] Serrano NC, Casas Juan P, Díaz Luis A, Páez Carolina, Mesa Clara M, (2004) Endothelial NO synthase genotype and risk of preeclampsia: a multicenter case-control study. Hypertension 44(5): 702–707. 15364897 10.1161/01.HYP.0000143483.66701.ec

[28] Nassereddine S, Idrissi Hind Hassani, Habbal Rachida, Abouelfath Rhizlane, Korch Farah, (2018) The polymorphism G894 T of endothelial nitric oxide synthase (eNOS) gene is associated with susceptibility to essential hypertension (EH) in Morocco. BMC Med Genet 19(1): 127. 30053839 10.1186/s12881-018-0638-1 PMC6062870

[29] Persu A, Vinck Wouter J, Khattabi Ouarda El, Janssen Rob G J H, Paulussen Aimée D C, (2005) Influence of the endothelial nitric oxide synthase gene on conventional and ambulatory blood pressure: sib-pair analysis and haplotype study. J Hypertens 23(4): 759–765. 15775780 10.1097/01.hjh.0000163144.74588.ad

[30] Chenou F, Albuquerque DM, Leonardo DP, Domingos IF, Bezerra MAC, (2020) Endothelial Nitric Oxide Synthase (eNOS) Gene Polymorphisms and Markers of Hemolysis, Inflammation and Endothelial Dysfunction in Brazilian Sickle Cell Anemia Patients. Biochem Genet 58(4): 580–594. 32277314 10.1007/s10528-020-09959-w

[31] Kourosh Arami M, Hosseini N, Mohsenzadegan M, Komaki A, Joghataei MT (2020) Neurophysiologic implications of neuronal nitric oxide synthase. Rev Neurosci 31(6): 617–636. 32739909 10.1515/revneuro-2019-0111

[32] Pautz A, Li H, Kleinert H (2021) Regulation of NOS expression in vascular diseases. Front Biosci (Landmark Ed) 26(5): 85–101. 34027652 10.52586/4926

[33] Francis BN, Salameh M, Khamisy Farah R, Farah R (2018) Tetrahydrobiopterin (BH4 ): Targeting endothelial nitric oxide synthase as a potential therapy for pulmonary hypertension. Cardiovasc Ther 36(1). 10.1111/1755-5922.12312 29151278

[34] Rafikov R, Fonseca Fabio V, Kumar Sanjiv, Pardo Daniel, Darragh Charles, (2011) eNOS activation and NO function: structural motifs responsible for the posttranslational control of endothelial nitric oxide synthase activity. J Endocrinol 210(3): 271–284. 21642378 10.1530/JOE-11-0083 PMC3326601

[35] Crabtree MJ, Smith CL, Lam G, Goligorsky MS, Gross SS (2008) Ratio of 5,6,7,8-tetrahydrobiopterin to 7,8-dihydrobiopterin in endothelial cells determines glucose-elicited changes in NO vs. superoxide production by eNOS. Am J Physiol Heart Circ Physiol 294(4): H1530–1540. 18192221 10.1152/ajpheart.00823.2007 PMC2722919

[36] Vasquez Vivar J, Martasek P, Whitsett J, Joseph J, Kalyanaraman B (2002) The ratio between tetrahydrobiopterin and oxidized tetrahydrobiopterin analogues controls superoxide release from endothelial nitric oxide synthase: an EPR spin trapping study. Biochem J 362(Pt 3): 733–739. 11879202 10.1042/0264-6021:3620733 PMC1222439

[37] Zhao Y, Vanhoutte PM, Leung SW (2015) Vascular nitric oxide: Beyond eNOS. J Pharmacol Sci 129(2): 83–94. 26499181 10.1016/j.jphs.2015.09.002

[38] Maron BA, Michel T (2012) Subcellular localization of oxidants and redox modulation of endothelial nitric oxide synthase. Circ J 76(11): 2497–2512. 23075817 10.1253/circj.cj-12-1207

[39] Gebhart V, Reiss K, Kollau A, Mayer B, Gorren ACF (2019) Site and mechanism of uncoupling of nitric-oxide synthase: Uncoupling by monomerization and other misconceptions. Nitric Oxide 89:14–21. 31022534 10.1016/j.niox.2019.04.007

[40] Garcia V, Sessa WC (2019) Endothelial NOS: perspective and recent developments. Br J Pharmacol 176(2) 189–196. 30341769 10.1111/bph.14522 PMC6295413

[41] Daiber A, Xia Ning, Steven Sebastian, Oelze Matthias, Hanf Alina, (2019) New Therapeutic Implications of Endothelial Nitric Oxide Synthase (eNOS) Function/Dysfunction in Cardiovascular Disease. Int J Mol Sci 20(1): 187. 10.3390/ijms20010187 PMC6337296 30621010

[42] Kawashima S (2004) Malfunction of vascular control in lifestyle-related diseases: endothelial nitric oxide (NO) synthase/NO system in atherosclerosis. J Pharmacol Sci 96(4): 411–419. 15613778 10.1254/jphs.fmj04006x6

[43] Heitzer T, Schlinzig T, Krohn K, Meinertz T, Munzel T (2001) Endothelial dysfunction, oxidative stress, and risk of cardiovascular events in patients with coronary artery disease. Circulation 104(22): 2673–2678. 11723017 10.1161/hc4601.099485

[44] Mozos I, Luca CT (2017) Crosstalk between Oxidative and Nitrosative Stress and Arterial Stiffness. Curr Vasc Pharmacol 15(5): 446–456. 28155616 10.2174/1570161115666170201115428

[45] Opatrilova R, Kubatka Peter, Caprnda Martin, Büsselberg Dietrich, Krasnik Vladimir, (2018) Nitric oxide in the pathophysiology of retinopathy: Evidences from preclinical and clinical researches. Acta Ophthalmol 96(3): 222–231. 28391624 10.1111/aos.13384

[46] Cave AC, Brewer Alison C, Narayanapanicker Anilkumar, Ray Robin, Grieve David J, (2006) NADPH oxidases in cardiovascular health and disease. Antioxid Redox Signal 8(5-6): 691–728. 16771662 10.1089/ars.2006.8.691

[47] Guzik TJ, Hoch Nyssa E, Brown Kathryn A, McCann Louise A, Rahman Ayaz, (2007) Role of the T cell in the genesis of angiotensin II induced hypertension and vascular dysfunction. J Exp Med 204(10): 2449–2460. 17875676 10.1084/jem.20070657 PMC2118469

[48] Mikhed Y, Daiber A, Steven S (2015) Mitochondrial Oxidative Stress, Mitochondrial DNA Damage and Their Role in Age-Related Vascular Dysfunction. Int J Mol Sci 16(7): 15918–15953. 26184181 10.3390/ijms160715918 PMC4519931

[49] Katseff A, Alhawaj R, Wolin MS (2021) Redox and Inflammatory Signaling, the Unfolded Protein Response, and the Pathogenesis of Pulmonary Hypertension. Adv Exp Med Biol 1304: 333–373. 34019276 10.1007/978-3-030-68748-9_17

[50] Channon KM (2021) Tetrahydrobiopterin and Nitric Oxide Synthase Recouplers. Handb Exp Pharmacol 264: 339–352. 33140223 10.1007/164_2020_390

[51] Allerton TD, Proctor David N, Stephens Jacqueline M, Dugas Tammy R, Spielmann Guillaume, (2018) l-Citrulline Supplementation: Impact on Cardiometabolic Health. Nutrients 10(7): 921. 10.3390/nu10070921 PMC6073798 30029482

[52] Mirenayat MS, Moradi S, Mohammadi H, Rouhani MH (2018) Effect of L-Citrulline Supplementation on Blood Pressure: a Systematic Review and Meta-Analysis of Clinical Trials. Curr Hypertens Rep 20: 98. 30284051 10.1007/s11906-018-0898-3

[53] Khalaf D, Kruger M, Wehland M, Infanger M, Grimm D (2019) The Effects of Oral l-Arginine and l-Citrulline Supplementation on Blood Pressure. Nutrients 11(7): 1679. 10.3390/nu11071679 PMC6683098 31336573

[54] Barkhidarian B, Khorshidi M, ShabBidar S, Hashemi B (2019) Effects of L-citrulline supplementation on blood pressure: A systematic review and meta-analysis. Avicenna J Phytomed 9(1): 10–20. 30788274 PMC6369322

[55] Gonzalez AM, Trexler ET (2020) Effects of Citrulline Supplementation on Exercise Performance in Humans: A Review of the Current Literature. J Strength Cond Res 34(5): 1480–1495. 31977835 10.1519/JSC.0000000000003426

[56] Trexler ET, Persky Adam M, Ryan Eric D, Schwartz Todd A, Stoner 1 Lee, (2019) Acute Effects of Citrulline Supplementation on High-Intensity Strength and Power Performance: A Systematic Review and Meta-Analysis. Sports Med 49(5): 707–718. 30895562 10.1007/s40279-019-01091-z

[57] Yang Y, Liu Song, Fan Zhenhua, Li Zhuo, Liu Jing, (2014) Sp1 modification of human endothelial nitric oxide synthase promoter increases the hypoxia-stimulated activity. Microvasc Res 93: 80–86. 24681424 10.1016/j.mvr.2014.03.004

[58] Karantzoulis Fegaras F, Antoniou H, Lai SL, Kulkarni G, D’Abreo C, (1999) Characterization of the human endothelial nitric-oxide synthase promoter. J Biol Chem 274(5): 3076–3093. 9915847 10.1074/jbc.274.5.3076

[59] Caviedes Ariel, Maturana Barbara, Corvalán Katherina, Engler Alexander, Gordillo Felipe, (2021) eNOS-dependent S-nitrosylation of the NF-kappaB subunit p65 has neuroprotective effects. Cell Death Dis 12: 4. 33414434 10.1038/s41419-020-03338-4 PMC7790835

[60] Drummond GR, Cai H, Davis ME, Ramasamy S, Harrison DG (2000) Transcriptional and posttranscriptional regulation of endothelial nitric oxide synthase expression by hydrogen peroxide. Circ Res 86(3): 347–354. 10679488 10.1161/01.res.86.3.347

[61] Searles CD (2006) Transcriptional and posttranscriptional regulation of endothelial nitric oxide synthase expression. Am J Physiol Cell Physiol 291(5): C803–816. 16738003 10.1152/ajpcell.00457.2005

[62] Ju H, Zou R, Venema VJ, Venema RC (1997) Direct interaction of endothelial nitric-oxide synthase and caveolin-1 inhibits synthase activity. J Biol Chem 272(30): 18522–18525. 9228013 10.1074/jbc.272.30.18522

[63] Qian J, Fulton D (2013) Post-translational regulation of endothelial nitric oxide synthase in vascular endothelium. Front Physiol 4: 347. 24379783 10.3389/fphys.2013.00347 PMC3861784

[64] Rajapakse NW, Karim F, Straznicky NE, Fernandez S, Evans RG, (2014) Augmented endothelial-specific L-arginine transport prevents obesity-induced hypertension. Acta Physiol (Oxf) 212(1): 39–48. 25041756 10.1111/apha.12344

[65] Zhang Y, Janssens SP, Wingler K, Schmidt HH, Moens AL (2011) Modulating endothelial nitric oxide synthase: a new cardiovascular therapeutic strategy. Am J Physiol Heart Circ Physiol 301(3): H634–646. 21622818 10.1152/ajpheart.01315.2010

[66] Pala R, Mohieldin Ashraf M, Sherpa Rinzhin T, Kathem Sarmed H, Shamloo Kiumars, (2019) Ciliotherapy: Remote Control of Primary Cilia Movement and Function by Magnetic Nanoparticles. ACS Nano 13(3): 3555–3572. 30860808 10.1021/acsnano.9b00033 PMC7899146

[67] Pala R, Mohieldin Ashraf M, Shamloo Kiumars, Sherpa Rinzhin T, Kathem Sarmed H, (2019) Personalized Nanotherapy by Specifically Targeting Cell Organelles To Improve Vascular Hypertension. Nano Lett 19(2): 904–914. 30582331 10.1021/acs.nanolett.8b04138 PMC7899193

[68] Kolluru GK, Sinha Swaraj, Majumder Syamantak, Muley Ajit, Siamwala Jamila H, (2010) Shear stress promotes nitric oxide production in endothelial cells by sub-cellular delocalization of eNOS: A basis for shear stress mediated angiogenesis. Nitric Oxide 22(4): 304–315. 20188204 10.1016/j.niox.2010.02.004

[69] AbouAlaiwi WA, Takahashi Maki, Mell Blair R, Jones Thomas J, Ratnam Shobha, (2009) Ciliary polycystin-2 is a mechanosensitive calcium channel involved in nitric oxide signaling cascades. Circ Res 104(7): 860–869. 19265036 10.1161/CIRCRESAHA.108.192765 PMC3085025

[70] Nauli SM, Kawanabe Yoshifumi, Kaminski John J, Pearce William J, Ingber Donald E, (2008) Endothelial cilia are fluid shear sensors that regulate calcium signaling and nitric oxide production through polycystin-1. Circulation 117(9): 1161–1171. 18285569 10.1161/CIRCULATIONAHA.107.710111 PMC3071982

[71] Miyazaki Akita A, Hayashi Toshio, Ding Qun Fang, Shiraishi Hiroaki, Nomura Takahide, (2007) 17beta-estradiol antagonizes the down-regulation of endothelial nitric-oxide synthase and GTP cyclohydrolase I by high glucose: relevance to postmenopausal diabetic cardiovascular disease. J Pharmacol Exp Ther 320(2): 591–598. 17082313 10.1124/jpet.106.111641

[72] Coppo R, Amore A (2000) Importance of the bradykinin-nitric oxide synthase system in the hypersensitivity reactions of chronic haemodialysis patients. Nephrol Dial Transplant 15(9): 1288–1290. 10978379 10.1093/ndt/15.9.1288

[73] Sessa WC (2004) eNOS at a glance. J Cell Sci 117( Pt 12): 2427–2429. 15159447 10.1242/jcs.01165

[74] Chen J, Shi Minmin, Wang Na, Yi Pengfei, Sun Lin, (2019) TSH inhibits eNOS expression in HMEC-1 cells through the TSHR/PI3K/AKT signaling pathway. Ann Endocrinol (Paris) 80(5–6): 273–279. 31606200 10.1016/j.ando.2019.06.007

[75] Thomas SR, Chen K, Keaney JF Jr (2002) Hydrogen peroxide activates endothelial nitric-oxide synthase through coordinated phosphorylation and dephosphorylation via a phosphoinositide 3-kinase-dependent signaling pathway. J Biol Chem 277(8): 6017–6024. 11744698 10.1074/jbc.M109107200

[76] Harris MB, Ju H, Venema VJ, Liang H, Zou R, (2001) Reciprocal phosphorylation and regulation of endothelial nitric-oxide synthase in response to bradykinin stimulation. J Biol Chem 276(19): 16587–16591. 11340086 10.1074/jbc.M100229200

[77] Heiss C, Rodriguez Mateos A, Kelm M (2015) Central role of eNOS in the maintenance of endothelial homeostasis. Antioxid Redox Signal 22(14): 1230–1242. 25330054 10.1089/ars.2014.6158 PMC4410282

[78] Escribano M, Molero Laura, Farré Antonio López, Abarrategui Cynthia, Carrasco, (2004) Aspirin inhibits endothelial nitric oxide synthase (eNOS) and Flk-1 (vascular endothelial growth factor receptor-2) prior to rat colon tumour development. Clin Sci (Lond) 106(1): 83–91. 12943528 10.1042/CS20030192

[79] Snoep JD, Hovens Marcel M C, Pasha Sharif M, Frölich Marijke, Pijl Hanno, (2009) Time-dependent effects of low-dose aspirin on plasma renin activity, aldosterone, cortisol, and catecholamines. Hypertension 54(5): 1136–1142. 19805643 10.1161/HYPERTENSIONAHA.109.134825

[80] Moriarty F, Ebell MH (2020) A comparison of contemporary versus older studies of aspirin for primary prevention. Fam Pract 37(3): 290–296. 31751455 10.1093/fampra/cmz080

[81] Namba T, Koike Hiromi, Murakami Kazushi, Aoki Motokuni, Makino Hirofumi, (2003) Angiogenesis induced by endothelial nitric oxide synthase gene through vascular endothelial growth factor expression in a rat hindlimb ischemia model. Circulation 108(18): 2250–2257. 14568906 10.1161/01.CIR.0000093190.53478.78

[82] Cho DH, Choi YJ, Jo SA, Jo I (2004) Nitric oxide production and regulation of endothelial nitric-oxide synthase phosphorylation by prolonged treatment with troglitazone: evidence for involvement of peroxisome proliferator-activated receptor (PPAR) gamma-dependent and PPARgamma-independent signaling pathways. J Biol Chem 279(4): 2499–2506. 14593122 10.1074/jbc.M309451200

[83] Evgenov OV, Pacher Pál, Schmidt Peter M, Haskó György, Schmidt Harald H H W, (2006) NO-independent stimulators and activators of soluble guanylate cyclase: discovery and therapeutic potential. Nat Rev Drug Discov 5(9): 755–768. 16955067 10.1038/nrd2038 PMC2225477

[84] Laursen JB, Somers M, Kurz S, McCann L, Warnholtz A, (2001) Endothelial regulation of vasomotion in apoE-deficient mice: implications for interactions between peroxynitrite and tetrahydrobiopterin. Circulation 103(9): 1282–1288. 11238274 10.1161/01.cir.103.9.1282

[85] Wenzel P, Schulz Eberhard, Oelze Matthias, Müller Johanna, Schuhmacher Swenja, (2008) AT1-receptor blockade by telmisartan upregulates GTP-cyclohydrolase I and protects eNOS in diabetic rats. Free Radic Biol Med 45(5): 619–626. 18539157 10.1016/j.freeradbiomed.2008.05.009

[86] Wenzel P, Daiber Andreas, Oelze Matthias, Brandt Moritz, Closs Ellen, (2008) Mechanisms underlying recoupling of eNOS by HMG-CoA reductase inhibition in a rat model of streptozotocin-induced diabetes mellitus. Atherosclerosis 198(1): 65–76. 18061195 10.1016/j.atherosclerosis.2007.10.003 PMC2889614

[87] Su KH, Tsai Jin Yi, Kou Yu Ru, Chiang An Na, Hsiao Sheng Huang, (2009) Valsartan regulates the interaction of angiotensin II type 1 receptor and endothelial nitric oxide synthase via Src/PI3K/Akt signalling. Cardiovasc Res 82(3): 468–475. 19307231 10.1093/cvr/cvp091

[88] Ding J, Yu Min, Jiang Juncai, Luo Yanbei, Zhang Qian, (2020) Angiotensin II Decreases Endothelial Nitric Oxide Synthase Phosphorylation via AT1R Nox/ROS/PP2A Pathway. Front Physiol 11: 566410. 33162896 10.3389/fphys.2020.566410 PMC7580705

[89] Li HM, Mo Zhi Wei, Peng Yue Ming, Li Yan, Dai Wei Ping, (2020) Angiogenic and Antiangiogenic mechanisms of high density lipoprotein from healthy subjects and coronary artery diseases patients. Redox Biol 36:101642. 32863238 10.1016/j.redox.2020.101642 PMC7364160

[90] Nauli SM (2019) Cholesterol may not have a special place in kidneys. Am J Physiol Renal Physiol 317(5): F1169–F1170. 31532244 10.1152/ajprenal.00394.2019 PMC6879944

[91] da Motta NA, de Brito FC (2016) Cilostazol exerts antiplatelet and antiinflammatory effects through AMPK activation and NF-kB inhibition on hypercholesterolemic rats. Fundam Clin Pharmacol 30(4): 327–337. 26950185 10.1111/fcp.12195

[92] Feron O, Dessy C, Moniotte S, Desager JP, Balligand JL (1999) Hypercholesterolemia decreases nitric oxide production by promoting the interaction of caveolin and endothelial nitric oxide synthase. J Clin Invest 103(6): 897–905. 10079111 10.1172/JCI4829 PMC408139

[93] Andrade VL, Sertório JTC, Eleuterio NM, Tanus-Santos JE, Fernandes KS, (2013) Simvastatin treatment increases nitrite levels in obese women: modulation by T(−786)C polymorphism of eNOS. Nitric Oxide 33: 83–87. 23876348 10.1016/j.niox.2013.07.005

[94] Silva PS, Fontana V, Luizon MR, Lacchini R, Silva WA Jr, (2013) eNOS and BDKRB2 genotypes affect the antihypertensive responses to enalapril. Eur J Clin Pharmacol 69(2): 167–177. 22706620 10.1007/s00228-012-1326-2

[95] Lacchini R, Tanus Santos JE (2014) Pharmacogenetics of erectile dysfunction: navigating into uncharted waters. Pharmacogenomics 15(11): 1519–1538. 25303302 10.2217/pgs.14.110

[96] Moncada S, Higgs (1993) A The L-arginine-nitric oxide pathway. N Engl J Med 329(27): 2002–2012. 7504210 10.1056/NEJM199312303292706

[97] Jones SP, Girod WG, Palazzo AJ, Granger DN, Grisham MB, (1999) Myocardial ischemia-reperfusion injury is exacerbated in absence of endothelial cell nitric oxide synthase. Am J Physiol 276(5): H1567–1573. 10330240 10.1152/ajpheart.1999.276.5.H1567

[98] Brunner F, Maier Robert, Andrew Penelope, Wölkart Gerald, Zechner Rudolf, (2003) Attenuation of myocardial ischemia/reperfusion injury in mice with myocyte-specific overexpression of endothelial nitric oxide synthase. Cardiovasc Res 57(1): 55–62. 12504814 10.1016/s0008-6363(02)00649-1

[99] Champion HC, Georgakopoulos Dimitrios, Takimoto Eiki, Isoda Takayoshi, Wang Yibin, (2004) Modulation of in vivo cardiac function by myocyte-specific nitric oxide synthase-3. Circ Res 94(5): 657–663. 14752030 10.1161/01.RES.0000119323.79644.20

[100] Feron O, Belhassen L, Kobzik L, Smith TW, Kelly RA, (1996) Endothelial nitric oxide synthase targeting to caveolae. Specific interactions with caveolin isoforms in cardiac myocytes and endothelial cells. J Biol Chem 271(37): 22810–22814. 8798458 10.1074/jbc.271.37.22810

[101] Chen Z, Qi Y, Gao C (2015) Cardiac myocyte-protective effect of microRNA-22 during ischemia and reperfusion through disrupting the caveolin-3/eNOS signaling. Int J Clin Exp Pathol 8(5): 4614–4626. 26191152 PMC4503024

[102] Das A, Xi L, Kukreja RC (2005) Phosphodiesterase-5 inhibitor sildenafil preconditions adult cardiac myocytes against necrosis and apoptosis. Essential role of nitric oxide signaling. J Biol Chem 280(13): 12944–12955. 15668244 10.1074/jbc.M404706200

[103] Seya K, Ono Kyoichi, Fujisawa Susumu, Okumura Ken, Motomura Shigeru, (2013) Cytosolic Ca2+-induced apoptosis in rat cardiomyocytes via mitochondrial NO-cGMP-protein kinase G pathway. J Pharmacol Exp Ther 344(1): 77–84. 23104881 10.1124/jpet.112.198176

[104] Jang JH, Chun Jung Nyeo, Godo Shigeo, Wu Guangyu, Shimokawa Hiroaki, (2015) ROS and endothelial nitric oxide synthase (eNOS)-dependent trafficking of angiotensin II type 2 receptor begets neuronal NOS in cardiac myocytes. Basic Res Cardiol 110(3): 21. 25804308 10.1007/s00395-015-0477-6 PMC4827156

[105] Brede M, Roell Wilhelm, Ritter Oliver, Wiesmann Frank, Jahns Roland, (2003) Cardiac hypertrophy is associated with decreased eNOS expression in angiotensin AT2 receptor-deficient mice. Hypertension 42(6): 1177–1182. 14581297 10.1161/01.HYP.0000100445.80029.8E

[106] Sartoretto JL, Kalwa Hermann, Shiroto Takashi, Sartoretto Simone M, Pluth Michael D, (2012) Role of Ca2+ in the control of H2O2-modulated phosphorylation pathways leading to eNOS activation in cardiac myocytes. PLoS One 7(9): e44627. 22970272 10.1371/journal.pone.0044627 PMC3435284

[107] Yang R, Beqiri Dardan, Shen Jian Bing, Redden John M, Kafka Kimberly Dodge, (2015) P2X4 receptor-eNOS signaling pathway in cardiac myocytes as a novel protective mechanism in heart failure. Comput Struct Biotechnol J 13:1–7. 25750695 10.1016/j.csbj.2014.11.002 PMC4348440

[108] Stokes L, Bidula S, Bibic L, Alum E (2020) To Inhibit or Enhance? Is There a Benefit to Positive Allosteric Modulation of P2X Receptors? Front Pharmacol 11: 627. 32477120 10.3389/fphar.2020.00627 PMC7235284

[109] Gyurko R, Kuhlencordt P, Fishman MC, Huang PL (2000) Modulation of mouse cardiac function in vivo by eNOS and ANP. Am J Physiol Heart Circ Physiol 278(3): H971–981. 10710367 10.1152/ajpheart.2000.278.3.H971

[110] Massion PB, Balligand JL (2003) Modulation of cardiac contraction, relaxation and rate by the endothelial nitric oxide synthase (eNOS): lessons from genetically modified mice. J Physiol 546(Pt 1): 63–75. 12509479 10.1113/jphysiol.2002.025973 PMC2342468

[111] Kubis N, Besnard Sandrine, Silvestre Jean Sébastien, Feletou Michel, Huang Paul L, (2002) Decreased arteriolar density in endothelial nitric oxide synthase knockout mice is due to hypertension, not to the constitutive defect in endothelial nitric oxide synthase enzyme. J Hypertens 20(2): 273–280. 11821712 10.1097/00004872-200202000-00017

[112] Guo W, Kada K, Kamiya K, Toyama J (1997) IGF-I regulates K(+)-channel expression of cultured neonatal rat ventricular myocytes. Am J Physiol 272(6): H2599–2606. 9227536 10.1152/ajpheart.1997.272.6.H2599

[113] de Waard MC, van Haperen Rien, Soullié Thomas, Tempel Dennie, de Crom Rini, (2010) Beneficial effects of exercise training after myocardial infarction require full eNOS expression. J Mol Cell Cardiol 48(6): 1041–1049. 20153335 10.1016/j.yjmcc.2010.02.005

[114] Kawanabe Y, Takahashi Maki, Jin Xingjian, Majeed Shakila Abdul, Nauli Andromeda M, (2012) Cilostazol prevents endothelin-induced smooth muscle constriction and proliferation. PLoS One 7(9): e44476. 22957074 10.1371/journal.pone.0044476 PMC3434142

[115] Vignon Zellweger N, Relle Katharina, Kienlen Elodie, Alter Markus, Seider Patrick, (2011) Endothelin-1 overexpression restores diastolic function in eNOS knockout mice. J Hypertens 29(5): 961–970. 21451422 10.1097/HJH.0b013e3283450770

[116] Shibata K, Yatera Yasuko, Furuno Yumi, Sabanai Ken, Morisada Naoya, (2010) Spontaneous development of left ventricular hypertrophy and diastolic dysfunction in mice lacking all nitric oxide synthases. Circ J 74(12): 2681–2692. 20966596 10.1253/circj.cj-10-0277

[117] Nakata S, Tsutsui Masato, Shimokawa Hiroaki, Suda Osamu, Morishita Tsuyoshi, (2008) Spontaneous myocardial infarction in mice lacking all nitric oxide synthase isoforms. Circulation 117(17): 2211–2223. 18413498 10.1161/CIRCULATIONAHA.107.742692

[118] Lin X, Wang Qiongying, Sun Shougang, Xu Guangli, Wu Qiang (2020) Astragaloside IV promotes the eNOS/NO/cGMP pathway and improves left ventricular diastolic function in rats with metabolic syndrome. J Int Med Res 48(1): 300060519826848. 30843445 10.1177/0300060519826848 PMC7140221

[119] Martinelli NC, Santos Kátia G, Biolo Andréia, La Porta Vanessa L, Cohen Carolina R, (2012) Polymorphisms of endothelial nitric oxide synthase gene in systolic heart failure: an haplotype analysis. Nitric Oxide 26(3): 141–147. 22290017 10.1016/j.niox.2012.01.003

[120] Li J, Zhang Yangyang, Li Chuanfu, Xie Jian, Liu Ying, (2013) HSPA12B attenuates cardiac dysfunction and remodelling after myocardial infarction through an eNOS-dependent mechanism. Cardiovasc Res 99(4): 674–684. 23729663 10.1093/cvr/cvt139

[121] Fukushima H, Kobayashi N, Takeshima H, Koguchi W, Ishimitsu T (2010) Effects of olmesartan on Apelin/APJ and Akt/endothelial nitric oxide synthase pathway in Dahl rats with end-stage heart failure. J Cardiovasc Pharmacol 55(1): 83–88. 19904215 10.1097/FJC.0b013e3181c87a82

[122] Feil R, Lehners M, Stehle D, Feil S (2021) Visualising and understanding cGMP signals in the cardiovascular system. Br J Pharmacol. 10.1111/bph.15500 33880767

[123] Perrien DS, Saleh Mohamed A, Takahashi Keiko, Madhur Meena S, Harrison David G, (2016) Novel methods for microCT-based analyses of vasculature in the renal cortex reveal a loss of perfusable arterioles and glomeruli in eNOS−/− mice. BMC Nephrol 17: 24. 26936597 10.1186/s12882-016-0235-5 PMC4776352

[124] Fulton D, Papapetropoulos A, Zhang X, Catravas JD, Hintze TH, (2000) Quantification of eNOS mRNA in the canine cardiac vasculature by competitive PCR. Am J Physiol Heart Circ Physiol 278(2): H658–665. 10666099 10.1152/ajpheart.2000.278.2.H658

[125] Lauer T, Kleinbongard Petra, Preik Michael, Rauch Bernhard H, Deussen Andreas, (2003) Direct biochemical evidence for eNOS stimulation by bradykinin in the human forearm vasculature. Basic Res Cardiol 98(2): 84–89. 12607129 10.1007/s003950300000

[126] Laughlin MH, Pollock JS, Amann JF, Hollis ML, Woodman CR, (2001) Training induces nonuniform increases in eNOS content along the coronary arterial tree. J Appl Physiol (1985) 90(2): 501–510. 11160048 10.1152/jappl.2001.90.2.501

[127] Yang S, Bae L, Zhang L (2000) Estrogen increases eNOS and NOx release in human coronary artery endothelium. J Cardiovasc Pharmacol 36(2): 242–247. 10942167 10.1097/00005344-200008000-00015

[128] Couto GK, Paula SM, Gomes Santos IL, Negrao CE, Rossoni LV (2018) Exercise training induces eNOS coupling and restores relaxation in coronary arteries of heart failure rats. Am J Physiol Heart Circ Physiol 314(4): H878–H887. 29351461 10.1152/ajpheart.00624.2017

[129] Venugopal SK, Devaraj S, Yuhanna I, Shaul P, Jialal I (2002) Demonstration that C-reactive protein decreases eNOS expression and bioactivity in human aortic endothelial cells. Circulation 106(12): 1439–1441. 12234944 10.1161/01.cir.0000033116.22237.f9

[130] Ito Y, Ohkubo Takeshi, Asano Yoshio, Hattori Kimihiko, Shimazu Tomokazu, (2010) Nitric oxide production during cerebral ischemia and reperfusion in eNOS- and nNOS-knockout mice. Curr Neurovasc Res 7(1): 23–31. 20158465 10.2174/156720210790820190

[131] Aoki T, Nishimura Masaki, Kataoka Hiroharu, Ishibashi Ryota, Nozaki Kazuhiko, (2011) Complementary inhibition of cerebral aneurysm formation by eNOS and nNOS. Lab Invest 91: 619–626. 21321533 10.1038/labinvest.2010.204

[132] Nan W, Zhonghang Xu, Keyan Chen, Tongtong Liu, Wanshu Guo, (2018) Epigallocatechin-3-Gallate Reduces Neuronal Apoptosis in Rats after Middle Cerebral Artery Occlusion Injury via PI3K/AKT/eNOS Signaling Pathway. Biomed Res Int 2018: 6473580. 29770336 10.1155/2018/6473580 PMC5889863

[133] Asahi M, Huang Zhihong, Thomas Sunu, Yoshimura Shin-ichi, Sumii Toshihisa, (2005) Protective effects of statins involving both eNOS and tPA in focal cerebral ischemia. J Cereb Blood Flow Metab 25(6): 722–729. 15716855 10.1038/sj.jcbfm.9600070 PMC2742229

[134] Liu H, Liu Xiaoqian, Wei Xinbing, Chen Lin, Xiang Yanxiao, (2012) Losartan, an angiotensin II type 1 receptor blocker, ameliorates cerebral ischemia-reperfusion injury via PI3K/Akt-mediated eNOS phosphorylation. Brain Res Bull 89(1–2): 65–70. 22766267 10.1016/j.brainresbull.2012.06.010

[135] Enkhjargal B, Malaguit Jay, Ho Wing M, Jiang Wu, Wan Weifeng, (2019) Vitamin D attenuates cerebral artery remodeling through VDR/AMPK/eNOS dimer phosphorylation pathway after subarachnoid hemorrhage in rats. J Cereb Blood Flow Metab 39(2): 272–284. 28825325 10.1177/0271678X17726287 PMC6365598

[136] McCarty MF, DiNicolantonio JJ, O’Keefe JH (2015) Capsaicin may have important potential for promoting vascular and metabolic health. Open Heart 2(1): e000262. 26113985 10.1136/openhrt-2015-000262 PMC4477151

[137] Wang Y, Cui Lin, Xu Hui, Liu Suxiao, Zhu Feiyun, (2017) TRPV1 agonism inhibits endothelial cell inflammation via activation of eNOS/NO pathway. Atherosclerosis 260: 13–19. 28324760 10.1016/j.atherosclerosis.2017.03.016

[138] Tan XL, Xue Yue Qiang, Ma Tao, Wang Xiaofang, Li Jing Jing, (2015) Partial eNOS deficiency causes spontaneous thrombotic cerebral infarction, amyloid angiopathy and cognitive impairment. Mol Neurodegener 10: 24. 26104027 10.1186/s13024-015-0020-0 PMC4479241

[139] Ally A, Maher TJ (2011) Transient middle cerebral artery occlusion and reperfusion alters inducible NOS expression within the ventrolateral medulla and modulates cardiovascular function during static exercise. Can J Physiol Pharmacol 89(9): 639–646. 21851181 10.1139/y11-064

[140] Broos K, Feys HB, De Meyer SF, Vanhoorelbeke K, Deckmyn H (2011) Platelets at work in primary hemostasis. Blood Rev 25(4): 155–167. 21496978 10.1016/j.blre.2011.03.002

[141] Toda N, Ayajiki K, Okamura T (2009) Cerebral blood flow regulation by nitric oxide in neurological disorders. Can J Physiol Pharmacol 87(8): 581–594. 19767882 10.1139/y09-048

[142] Pautz A, Art Julia, Hahn Susanne, Nowag Sebastian, Voss Cornelia, (2010) Regulation of the expression of inducible nitric oxide synthase. Nitric Oxide 23(2): 75–93. 20438856 10.1016/j.niox.2010.04.007

[143] Tieu K, Ischiropoulos H, Przedborski S (2003) Nitric oxide and reactive oxygen species in Parkinson’s disease. IUBMB Life 55(6): 329–335. 12938735 10.1080/1521654032000114320

[144] Lee PC, Salyapongse AN, Bragdon GA, Shears LL 2nd, Watkins SC, (1999) Impaired wound healing and angiogenesis in eNOS-deficient mice. Am J Physiol 277(4): H1600–1608. 10516200 10.1152/ajpheart.1999.277.4.H1600

[145] Huang PL, Huang Z, Mashimo H, Bloch KD, Moskowitz MA, (1995) Hypertension in mice lacking the gene for endothelial nitric oxide synthase. Nature 377(6546): 239–242. 7545787 10.1038/377239a0

[146] Park HJ, Shin Jin Young, Kim Ha Na, Oh Se Hee, Song Sook K, (2015) Mesenchymal stem cells stabilize the blood-brain barrier through regulation of astrocytes. Stem Cell Res Ther 6: 187. 26420371 10.1186/s13287-015-0180-4 PMC4588687

[147] Araujo CV, Estato V, Tibiriçá E, Bozza PT, Castro Faria Neto HC, (2012) PPAR gamma activation protects the brain against microvascular dysfunction in sepsis. Microvasc Res 84(2): 218–221. 22659381 10.1016/j.mvr.2012.05.006

[148] Hirooka Y, Kishi T, Sakai K, Takeshita A, Sunagawa K (2011) Imbalance of central nitric oxide and reactive oxygen species in the regulation of sympathetic activity and neural mechanisms of hypertension. Am J Physiol Regul Integr Comp Physiol 300(4): R818–826. 21289238 10.1152/ajpregu.00426.2010

[149] Huang CC, Chan SH, Hsu KS (2003) cGMP/protein kinase G-dependent potentiation of glutamatergic transmission induced by nitric oxide in immature rat rostral ventrolateral medulla neurons in vitro. Mol Pharmacol 64(2): 521–532. 12869658 10.1124/mol.64.2.521

[150] Kishi T, Hirooka Yoshitaka, Kimura Yoshikuni, Sakai Koji, Ito Koji, (2003) Overexpression of eNOS in RVLM improves impaired baroreflex control of heart rate in SHRSP. Rostral ventrolateral medulla. Stroke-prone spontaneously hypertensive rats. Hypertension 41(2): 255–260. 12574091 10.1161/01.hyp.0000050649.30821.cb

[151] Smith, Barron KW (1990) Gabaergic responses in ventrolateral medulla in spontaneously hypertensive rats. Am J Physiol 258(2): 450–456. 10.1152/ajpregu.1990.258.2.R450 1968724

[152] Ishide T, Preuss CV, Maher TJ, Ally A (2005) Neurochemistry within ventrolateral medulla and cardiovascular effects during static exercise following eNOS antagonism. Neurosci Res 52(1): 21–30. 15811549 10.1016/j.neures.2005.01.002

[153] Thomas GD (2011) Neural control of the circulation. Adv Physiol Educ 35(1): 28–32. 21385998 10.1152/advan.00114.2010

[154] Sheng Y, Zhu L (2018) The crosstalk between autonomic nervous system and blood vessels. Int J Physiol Pathophysiol Pharmacol 10(1): 17–28. 29593847 PMC5871626

[155] Biancardi VC, Sook JS, Patrick MS, Hong Z, Kaushik PP, (2011) Contribution of central nervous system endothelial nitric oxide synthase to neurohumoral activation in heart failure rats. Hypertension 58(3): 454–463. 21825233 10.1161/HYPERTENSIONAHA.111.175810 PMC3189581

[156] Scheen AJ (2019) Effect of SGLT2 Inhibitors on the Sympathetic Nervous System and Blood Pressure. Curr Cardiol Rep 21(8): 70. 31227915 10.1007/s11886-019-1165-1

[157] Ally A (1998) Ventrolateral medullary control of cardiovascular activity during muscle contraction. Neurosci Biobehav Rev 23(1): 65–86. 9861613 10.1016/s0149-7634(97)00069-9

[158] Kishi T (2013) Regulation of the sympathetic nervous system by nitric oxide and oxidative stress in the rostral ventrolateral medulla: 2012 Academic Conference Award from the Japanese Society of Hypertension. Hypertens Res 36(10): 845–851. 23864055 10.1038/hr.2013.73

[159] Kneale BJ, Chowienczyk PJ, Brett SE, Coltart DJ, Ritter JM (2000) Gender differences in sensitivity to adrenergic agonists of forearm resistance vasculature. J Am Coll Cardiol 36(4): 1233–1238. 11028476 10.1016/s0735-1097(00)00849-4

[160] Seddon M, Narbeh M, Rafal D, Husain S, Benyu J, (2009) Effects of neuronal nitric oxide synthase on human coronary artery diameter and blood flow *in vivo*. Circulation 119(20): 2656–2662. 19433760 10.1161/CIRCULATIONAHA.108.822205

[161] Seddon MD, Chowienczyk PJ, Brett SE, Casadei B, Shah AM (2008) Neuronal nitric oxide synthase regulates basal microvascular tone in humans *in vivo*. Circulation 117(15): 1991–1996. 18391107 10.1161/CIRCULATIONAHA.107.744540

[162] Shabeeh H, Michael S, Sally B, Narbeh M, Barbara C, Ajay MS, (2013) Sympathetic activation increases NO release from eNOS but neither eNOS nor nNOS play an essential role in exercise hyperemia in the human forearm. Am J Physiol Heart Circ Physiol 304(9): 1225–1230. 10.1152/ajpheart.00783.2012 PMC3652092 23436331

[163] Kou R, Michel T (2007) Epinephrine regulation of the endothelial nitric-oxide synthase: roles of RAC1 and beta3-adrenergic receptors in endothelial NO signaling. J Biol Chem 282(45): 32719–32729. 17855349 10.1074/jbc.M706815200

[164] Barr LA, Jonathan PL, Yuuki S, Lili AB, Nawazish N, (2017) Exercise training provides cardioprotection by activating and coupling endothelial nitric oxide synthase via a beta3-adrenergic receptor-AMP-activated protein kinase signaling pathway. Med Gas Res 7(1): 1–8. 28480026 10.4103/2045-9912.202904 PMC5402342

[165] Binici Z, Mouridsen MR, Kober L, Sajadieh A (2011) Decreased nighttime heart rate variability is associated with increased stroke risk. Stroke 42(11): 3196–3201. 21921280 10.1161/STROKEAHA.110.607697

[166] Chapleau MW, Rotella DL, Reho JJ, Rahmouni K, Stauss HM (2016) Chronic vagal nerve stimulation prevents high-salt diet-induced endothelial dysfunction and aortic stiffening in stroke-prone spontaneously hypertensive rats. Am J Physiol Heart Circ Physiol 311(1): 276–285. 10.1152/ajpheart.00043.2016 PMC4967207 27208157

[167] Chataigneau T, Félétou M, Huang PL, Fishman MC, Duhault J, (1999) Acetylcholine-induced relaxation in blood vessels from endothelial nitric oxide synthase knockout mice. Br J Pharmacol 126(1): 219–226. 10051139 10.1038/sj.bjp.0702300 PMC1565804

[168] Cohen RA, Plane F, Najibi S, Huk I, Malinski T, (1997) Nitric oxide is the mediator of both endothelium-dependent relaxation and hyperpolarization of the rabbit carotid artery. Proc Natl Acad Sci U S A 94(8): 4193–4198. 9108128 10.1073/pnas.94.8.4193 PMC20600

[169] Giles TD, Sander GE, Nossaman BD, Kadowitz PJ (2012) Impaired vasodilation in the pathogenesis of hypertension: focus on nitric oxide, endothelial-derived hyperpolarizing factors, and prostaglandins. J Clin Hypertens (Greenwich) 14(4): 198–205. 22458740 10.1111/j.1751-7176.2012.00606.x PMC8108814

[170] Luscher TF, Diederich D, Weber E, Vanhoutte PM, Buhler FR (1988) Endothelium-dependent responses in carotid and renal arteries of normotensive and hypertensive rats. Hypertension 11(6): 573–578. 3260580 10.1161/01.hyp.11.6.573

[171] Kumada M, Terui N, Kuwaki T (1990) Arterial baroreceptor reflex: its central and peripheral neural mechanisms. Prog Neurobiol 35(3): 331–361. 2263735 10.1016/0301-0082(90)90036-g

[172] Stauss HM, Godecke A, Mrowka R, Schrader J, Persson PB (1999) Enhanced blood pressure variability in eNOS knockout mice. Hypertension 33(6): 1359–1363. 10373216 10.1161/01.hyp.33.6.1359

[173] Waki H, Sergey K, Liang FW, David M, Tsuyoshi S, (2003) Chronic inhibition of endothelial nitric oxide synthase activity in nucleus tractus solitarii enhances baroreceptor reflex in conscious rats. J Physiol 546(Pt 1): 233–242. 12509491 10.1113/jphysiol.2002.030270 PMC2342461

[174] Paton JF, Waki H, Abdala AP, Dickinson J, Kasparov S (2007) Vascular brain signaling in hypertension: role of angiotensin II and nitric oxide. Curr Hypertens Rep 9(3): 242–247. 17519132 10.1007/s11906-007-0043-1

[175] Smith SA, Mitchell JH, Li J (2005) Independent modification of baroreceptor and exercise pressor reflex function by nitric oxide in nucleus tractus solitarius. Am J Physiol Heart Circ Physiol 288(5): 2068–2076. 10.1152/ajpheart.00919.2003 15604127

[176] Meyrelles SS, Sharma RV, Mao HZ, Abboud FM, Chapleau MW (2003) Modulation of baroreceptor activity by gene transfer of nitric oxide synthase to carotid sinus adventitia. Am J Physiol Regul Integr Comp Physiol 284(5): 1190–1198. 10.1152/ajpregu.00735.2002 12676743

[177] Xing SY, Li YZ, Liu JX, Gai YQ, Chen ZH, (2010) Genetic influence on baroreflex sensitivity in normotensive young men. Am J Hypertens 23(6): 655–659. 20300066 10.1038/ajh.2010.30

[178] Nauli SM, Maher TJ, Pearce WJ, Ally A (2001) Effects of opioid receptor activation on cardiovascular responses and extracellular monoamines within the rostral ventrolateral medulla during static contraction of skeletal muscle. Neurosci Res 41(4): 373–383. 11755224 10.1016/s0168-0102(01)00296-6

[179] Nauli SM, Pearce WJ, Amer A, Maher TJ, Ally A (2001) Effects of nitric oxide and GABA interaction within ventrolateral medulla on cardiovascular responses during static muscle contraction. Brain Res 922(2): 234–242. 11743955 10.1016/s0006-8993(01)03177-8

[180] Towiwat P, Phattanarudee S, Maher TJ, Ally A (2015) Modulation of inducible nitric oxide synthase (iNOS) expression and cardiovascular responses during static exercise following iNOS antagonism within the ventrolateral medulla. Mol Cell Biochem 398(1–2): 185–194. 25234194 10.1007/s11010-014-2218-9

[181] Ally A, Kabadi S, Phattanarudee S, Patel M, Maher TJ (2007) Neuronal nitric oxide synthase (nNOS) blockade within the ventrolateral medulla differentially modulates cardiovascular responses and nNOS expression during static skeletal muscle contraction. Brain Res 1150: 21–31. 17382301 10.1016/j.brainres.2007.02.064

[182] Van Haperen R, Monique W, Elza D, Barend M, Michael K, (2002) Reduction of blood pressure, plasma cholesterol, and atherosclerosis by elevated endothelial nitric oxide. J Biol Chem 277(50): 48803–48807. 12364322 10.1074/jbc.M209477200

[183] Ally A, Maher TJ (2008) Endothelial NOS expression within the ventrolateral medulla can affect cardiovascular function during static exercise in stroked rats. Brain Res 1196: 33–40. 18234158 10.1016/j.brainres.2007.12.036

[184] Farah C, Michel LYM, Balligand JL (2018) Nitric oxide signalling in cardiovascular health and disease. Nat Rev Cardiol 15(5): 292–316. 29388567 10.1038/nrcardio.2017.224

[185] Nauli SM, Ally A, Zhang L, Gerthoffer WT, Pearce WJ (2000) Maturation attenuates the effects of cGMP on contraction, [Ca2+] i and Ca2+ sensitivity in ovine basilar arteries. Gen Pharmacol 35(2): 107–118. 11707317 10.1016/s0306-3623(01)00100-8

[186] Schlaich MP, Melinda MP, Belinda AA, Samara F, Tanneale M, (2004) Impaired L-arginine transport and endothelial function in hypertensive and genetically predisposed normotensive subjects. Circulation 110(24): 3680–3686. 15569830 10.1161/01.CIR.0000149748.79945.52

[187] Hishikawa K, Nakaki T, Suzuki H, Kato R, Saruta T (1993) Role of L-arginine-nitric oxide pathway in hypertension. J Hypertens 11(6): 639–645. 8397243 10.1097/00004872-199306000-00008

[188] Zhou L, Zhu DY (2009) Neuronal nitric oxide synthase: structure, subcellular localization, regulation, and clinical implications. Nitric Oxide 20(4): 223–230. 19298861 10.1016/j.niox.2009.03.001

[189] Cai H, Harrison DG (2000) Endothelial dysfunction in cardiovascular diseases: the role of oxidant stress. Circ Res 87(10): 840–844. 11073878 10.1161/01.res.87.10.840

[190] Virdis A, Alessandra B, Rocchina C, Emiliano D, Matteo F, (2013) Endothelial dysfunction in small arteries of essential hypertensive patients: role of cyclooxygenase-2 in oxidative stress generation. Hypertension 62(2): 337–344. 23734008 10.1161/HYPERTENSIONAHA.111.00995

[191] Wong WT, Tian XY, Huang Y (2013) Endothelial dysfunction in diabetes and hypertension: cross talk in RAS, BMP4, and ROS-dependent COX-2-derived prostanoids. J Cardiovasc Pharmacol 61(3): 204–214. 23232839 10.1097/FJC.0b013e31827fe46e

[192] Ferrario CM (2006) Role of angiotensin II in cardiovascular disease therapeutic implications of more than a century of research. J Renin Angiotensin Aldosterone Syst 7(1): 3–14. 17083068 10.3317/jraas.2006.003

[193] Gao L, Chalupsky K, Stefani E, Cai H (2009) Mechanistic insights into folic acid-dependent vascular protection: dihydrofolate reductase (DHFR)-mediated reduction in oxidant stress in endothelial cells and angiotensin II-infused mice: a novel HPLC-based fluorescent assay for DHFR activity. J Mol Cell Cardiol 47(6): 752–760. 19660467 10.1016/j.yjmcc.2009.07.025 PMC2784291

[194] Siu KL, Miao XN, Cai H (2014) Recoupling of eNOS with folic acid prevents abdominal aortic aneurysm formation in angiotensin II-infused apolipoprotein E null mice. PLoS One 9(2): e88899. 24558445 10.1371/journal.pone.0088899 PMC3928303

[195] Li S, Qian L, Xinyu L, Lin L, Weiwei Y, (2015) Aurantio-obtusin relaxes systemic arteries through endothelial PI3K/AKT/eNOS-dependent signaling pathway in rats. J Pharmacol Sci 128(3): 108–115. 26076958 10.1016/j.jphs.2015.05.006

[196] Kuzkaya N, Weissmann N, Harrison DG, Dikalov S (2003) Interactions of peroxynitrite, tetrahydrobiopterin, ascorbic acid, and thiols: implications for uncoupling endothelial nitric-oxide synthase. J Biol Chem 278(25): 22546–22554. 12692136 10.1074/jbc.M302227200

[197] Ulker S, McKeown PP, Bayraktutan U (2003) Vitamins reverse endothelial dysfunction through regulation of eNOS and NAD(P)H oxidase activities. Hypertension 41(3): 534–539. 12623955 10.1161/01.HYP.0000057421.28533.37

[198] Landmesser U, Sergey D, Russ SP, Louise MC, Tohru F, (2003) Oxidation of tetrahydrobiopterin leads to uncoupling of endothelial cell nitric oxide synthase in hypertension. J Clin Invest 111(8): 1201–1209. 12697739 10.1172/JCI14172 PMC152929

[199] Benjamin EJ, Paul M, Alvaro A, Marcio SB, Clifton WC, (2019) Heart disease and Stroke Statistics-2019 Update: A Report from the American Heart Association. Circulation 139(10): 56–528. 10.1161/CIR.0000000000000659 30700139

[200] Ohara Y, Peterson TE, Harrison DG (1993) Hypercholesterolemia increases endothelial superoxide anion production. J Clin Invest 91(6): 2546–2551. 8390482 10.1172/JCI116491 PMC443316

[201] Karbach S, Wenzel P, Waisman A, Munzel T, Daiber A (2014) eNOS uncoupling in cardiovascular diseases--the role of oxidative stress and inflammation. Curr Pharm Des 20(22): 3579–3594. 24180381 10.2174/13816128113196660748

[202] Doerries C, Karsten G, Denise HK, Maren L, Arnd S, (2007) Critical role of the NAD(P)H oxidase subunit p47phox for left ventricular remodeling/dysfunction and survival after myocardial infarction. Circ Res 100(6): 894–903. 17332431 10.1161/01.RES.0000261657.76299.ff

[203] Matsuno K, Hiroyuki Y, Kazumi I, Denan J, Masato K, (2005) Nox1 is involved in angiotensin II-mediated hypertension: a study in Nox1-deficient mice. Circulation 112(17): 2677–2685. 16246966 10.1161/CIRCULATIONAHA.105.573709

[204] Wenzel P, Swenja S, Joachim K, Johanna M, Marcus H, (2008) Manganese superoxide dismutase and aldehyde dehydrogenase deficiency increase mitochondrial oxidative stress and aggravate age-dependent vascular dysfunction. Cardiovasc Res 80(20): 280–289. 18596060 10.1093/cvr/cvn182 PMC3937602

[205] Torzewski M, Viola O, Andrei LK, Matthias O, Andreas D, (2007) Deficiency of glutathione peroxidase-1 accelerates the progression of atherosclerosis in apolipoprotein E-deficient mice. Arterioscler Thromb Vasc Biol 27(4): 850–857. 17255533 10.1161/01.ATV.0000258809.47285.07

[206] Dikalova A, Roza C, Bernard L, Guangjie C, James MC, (2005) Nox1 overexpression potentiates angiotensin II-induced hypertension and vascular smooth muscle hypertrophy in transgenic mice. Circulation 112(17): 2668–2676. 16230485 10.1161/CIRCULATIONAHA.105.538934

[207] Sibylle V, Ley K (2010) Interleukin 17 in vascular inflammation. Cytokine Growth Factor Rev 21(6): 463–469. 21075042 10.1016/j.cytogfr.2010.10.003 PMC3005323

[208] Madhur MS, Heinrich EL, Louise AMC, Yoichiro I, Yelena B, (2010) Interleukin 17 promotes angiotensin II-induced hypertension and vascular dysfunction. Hypertension 55(2): 500–507. 20038749 10.1161/HYPERTENSIONAHA.109.145094 PMC2819301

[209] Calvert JW, Susheel G, Saurabh J, James JMG, William HB, (2008) Acute metformin therapy confers cardioprotection against myocardial infarction via AMPK-eNOS-mediated signaling. Diabetes 57(3): 696–705. 18083782 10.2337/db07-1098

[210] Wang W, Baoyu T, Jingrui C, Rui B, Xuran Z, (2018) An injectable conductive hydrogel encapsulating plasmid DNA-eNOs and ADSCs for treating myocardial infarction. Biomaterials 160: 69–81. 29396380 10.1016/j.biomaterials.2018.01.021

[211] Chen LL, Yin H, Huang J (2007) Inhibition of TGF-beta1 signaling by eNOS gene transfer improves ventricular remodeling after myocardial infarction through angiogenesis and reduction of apoptosis. Cardiovasc Pathol 16(4): 221–230. 17637430 10.1016/j.carpath.2007.02.007

[212] Feng L, Jiling R, Yafei L, Guifang Y, Licheng K, (2019) Resveratrol protects against isoproterenol induced myocardial infarction in rats through VEGF-B/AMPK/eNOS/NO signalling pathway. Free Radio Res 53(1): 82–93. 10.1080/10715762.2018.1554901 30526144

[213] Niu X, Lianyou Z, Xue L, Yusheng X, Bin W, (2014) beta3-Adrenoreceptor stimulation protects against myocardial infarction injury via eNOS and nNOS activation. PLoS One 9(6): e98713. 24911015 10.1371/journal.pone.0098713 PMC4049583

[214] Sadasivan S, Maher TJ, Quang LS (2006) Gamma-Hydroxybutyrate (GHB), gamma-butyrolactone (GBL), and 1,4-butanediol (1,4-BD) reduce the volume of cerebral infarction in rodent transient middle cerebral artery occlusion. Ann N Y Acad Sci 1074: 537–544. 17105951 10.1196/annals.1369.054

[215] Morikawa E, (1994) L-arginine infusion promotes nitric oxide-dependent vasodilation, increases regional cerebral blood flow, and reduces infarction volume in the rat. Stroke 25(2): 429–435. 7508154 10.1161/01.str.25.2.429

[216] Mason RP, Ruslan K, Gehan H, Robert FJ, Charles AD, (2008) Synergistic effect of amlodipine and atorvastatin in reversing LDL-induced endothelial dysfunction. Pharm Res 25(8): 1798–1806. 18087679 10.1007/s11095-007-9491-1

[217] Szolnoki Z, Havasi V, Bene J, Komlósi K, Szöke D, (2005) Endothelial nitric oxide synthase gene interactions and the risk of ischaemic stroke. Acta Neurol Scand 111(1): 29–33. 15595935 10.1111/j.1600-0404.2004.00345.x

[218] Gertz K, Josef P, Golo K, Klaus BF, Benjamin W, (2006) Physical activity improves long-term stroke outcome via endothelial nitric oxide synthase-dependent augmentation of neovascularization and cerebral blood flow. Circ Res 99(10): 1132–1140 (2006). 17038638 10.1161/01.RES.0000250175.14861.77

[219] Harris RA, Padilla J, Hanlon KP, Rink LD, Wallace JP (2008) The flow-mediated dilation response to acute exercise in overweight active and inactive men. Obesity (Silver Spring) 16(3): 578–584. 18239561 10.1038/oby.2007.87

[220] Yamakawa H, Jezova M, Ando H, Saavedra JM (2003) Normalization of endothelial and inducible nitric oxide synthase expression in brain microvessels of spontaneously hypertensive rats by angiotensin II AT1 receptor inhibition. J Cereb Blood Flow Metab 23(3): 371–380. 12621312 10.1097/01.WCB.0000047369.05600.03

[221] Nishimura Y, Ito T, Saavedra JM (2000) Angiotensin II AT (1) blockade normalizes cerebrovascular autoregulation and reduces cerebral ischemia in spontaneously hypertensive rats. Stroke 31(10): 2478–2486. 11022082 10.1161/01.str.31.10.2478

[222] Gertz K, Ulrich L, Lindauer U, Georg N, Michael B, (2003) Withdrawal of statin treatment abrogates stroke protection in mice. Stroke 34(2): 551–557. 12574574 10.1161/01.str.0000054055.28435.bf

[223] Dimaio TA, Shoujian W, Qiong H, Elizabeth AS, Christine MS, (2008) Attenuation of retinal vascular development and neovascularization in PECAM-1-deficient mice. Dev Biol 315(1): 72–88. 18206868 10.1016/j.ydbio.2007.12.008 PMC2275901

[224] Sehgal SN, Baker H, Vezina C (1975) Rapamycin (AY-22,989), a new antifungal antibiotic. II. Fermentation, isolation and characterization. J Antibiot (Tokyo) 28(10): 727–732. 1102509 10.7164/antibiotics.28.727

[225] Beard DJ, Zhaojin L, Anna MS, Yvonne C, Marilyn JC, (2020) Rapamycin Induces an eNOS (Endothelial Nitric Oxide Synthase) Dependent Increase in Brain Collateral Perfusion in Wistar and Spontaneously Hypertensive Rats. Stroke 51(9): 2834–2843. 32772681 10.1161/STROKEAHA.120.029781 PMC7484020

